# Loss of plastid *ndh* genes in an autotrophic desert plant

**DOI:** 10.1016/j.csbj.2023.10.023

**Published:** 2023-10-14

**Authors:** Ardashir Kharabian-Masouleh, Agnelo Furtado, Bader Alsubaie, Othman Al-Dossary, Alex Wu, Ibrahim Al-Mssalem, Robert Henry

**Affiliations:** a Queensland Alliance for Innovation in Food and Agriculture (QAAFI), The University of Queensland, Carmody Rd, St Lucia, QLD 4072, Australia; b ARC Centre of Excellence for Plant Success in Nature and Agriculture, The University of Queensland, Carmody Rd, St Lucia, QLD 4072, Australia; c College of Agriculture and Food Sciences, King Faisal University (KFU), Al Hofuf, 36362 Saudi Arabia

**Keywords:** Jojoba, Plastome, *ndh*, Loss

## Abstract

Plant plastid genomes are highly conserved with most flowering plants having the same complement of essential plastid genes. Here, we report the loss of five of the eleven NADH dehydrogenase subunit genes (*ndh*) in the plastid of a desert plant jojoba (*Simmondsia chinensis*). The plastid genome of jojoba was 156,496 bp with one large single copy region (LSC), a very small single copy region (SSC) and two expanded inverted repeats (IRA + IRB). The NADH dehydrogenase (NDH) complex is comprised of several protein subunits, encoded by the *ndh* genes of the plastome and the nucleus. The *ndh* genes are critical to the proper functioning of the photosynthetic electron transport chain and protection of plants from oxidative stress. Most plants are known to contain all eleven *ndh* genes. Plants with missing or defective *ndh* genes are often heterotrophs either due to their complete or holo- or myco- parasitic nature. Plants with a defective NDH complex, caused by the deletion/pseudogenisation of some or all the *ndh* genes, survive in milder climates suggesting the likely extinction of plant lineages lacking these genes under harsh climates. Interestingly, some autotrophic plants do exist without *ndh* gene/s and can cope with high or low light. This implies that these plants are protected from oxidative stress by mechanisms excluding *ndh* genes. Jojoba has evolved mechanisms to cope with a non-functioning NDH complex and survives in extreme desert conditions with abundant sunlight and limited water.

## Highlights

• The plastid in jojoba was found to have an unusual structure with a very small single copy region and larger inverted repeats.
• Five of the 11 plastid NADH dehydrogenase subunit genes (*ndh*) genes are lost.
• Other desert plants with some level of *ndh* gene loss were identified.
• Alternate mechanisms to negate the loss of the *ndh* genes may be required for survival in the desert with high light and low water availability.

## Graphical Abstract

**Figure:**
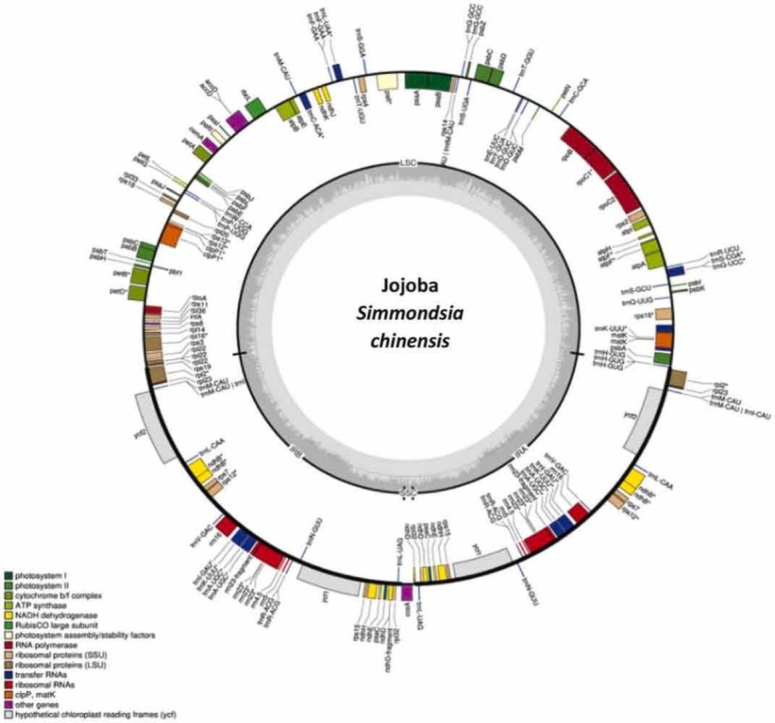
The plastome of Jojoba (*Simmondsia chinensis)* has lost five of the eleven *ndh* genes. In plants, the NDH complex encoded by the 11 *ndh* genes from the plastome is crucial for proper functioning of photosynthesis and protection from abiotic stress. A defective NDH complex from loss or pseudogenised *ndh* genes is dispensable to plants growing in environmentally milder conditions unless evolved to grow under harsh climates with heterotopic-adaptations. Jojoba and a few other exceptions show loss of the *ndh* genes and yet show no obvious heterotrophic adaptations to survive in harsh environments of extreme temperatures and water deficit. The loss of the *ndh* genes and the cyclic electron flow (CEF) in the photosynthetic machinery in jojoba, seems inconsequential on its survival. Further investigations are required to unravel the presence of an alternate CEF pathway known to be present in some plants and the presence and activity of crucial abiotic stress reducing metabolic reactions.

## 1 Introduction

The Earth’s oxygen rich atmosphere is thought to have started through the actions of oxygenic cyanobacteria-like prokaryotes around 2.5 billion years ago [1]. The cyanobacteria-like prokaryotes were autotrophic as they contained two photosystems to extract electrons from water, releasing oxygen and generating energy molecules for utility in food production [2], [3]. Plastids in plants are proposed to have descended from an ancient cyanobacterium-like prokaryote containing the two photosystems. About a billion or so years ago, an ancient cyanobacterium-like prokaryote was engulfed via endosymbiosis into a heterotrophic eukaryotic proteobacterium cell already containing a mitochondrion [4], [5], [6]. This process enabled the heterotrophic eukaryotic cell, with the engulfed cyanobacteria-like prokaryote, to evolve the ability to utilise photoenergy and transition from heterotrophy to autotrophy. The complete evolution of this proto-organelle into what are now known as plastids in plants, involved several changes including the loss of several hundreds or thousands of the original ancestral cyanobacteria genes. A small proportion of the cyanobacterial genes were retained (5–10%) with some integrated into the nucleus [7], [8], [9].

Plastids containing chlorophyll, known as chloroplasts [10] are semiautonomous organelles which are crucial to autotrophs for fixing atmospheric carbon in photosynthesis, where light energy is converted into chemical energy [11], [12], [13], [14]. Chloroplasts contain two light capture and reaction centres, photosystem II (PSII) and Photosystem I (PSI), linked in series for the linear flow of electrons (LEF), derived from water molecules to Nicotinamide adenine dinucleotide phosphate (NADPH) (reviewed in [15]). In LEF, PSII and PSI transport the excited electrons from water across the chloroplast thylakoid membrane in a linear electron transport chain (LET). Coupled to the LET is proton transfer, which generates an electrochemical gradient of protons to generate adenosine triphosphate (ATP) from PSII and NADPH from PSI. The ATP and NADPH are utilised as energy molecules to drive the reactions of the Benson-Calvin cycle to generate sugar molecules from organic carbon compounds produced by fixing CO_2_ molecules [16]. In the LEF, the main redox components or electron carriers involved with PSII are plastoquinone (PQ), cytochrome *b*_*6*_*f* complex (Cyt *b*_*6*_*f*), and plastocyanin (Pc), while those with the PSI are PSI reaction centre (P_700_) and ferredoxin (Fd) (reviewed in [15]).

In addition to the LET, the cyclic electron transport chain (CET) via cyclic flow of electrons (CEF), involves the excitation of PSI using electrons from PSII, but the electrons when transferred to Fd are not used to generate NADPH but recycled back to the PSII redox-intermediates completing the CEF and generation of ATP (reviewed in [15]). An important component of the CET is the NADH dehydrogenase (NDH) complex [17]. The NDH complex is comprised of several protein subunits, some encoded by *ndh* genes. Eleven of the *ndh* genes are encoded by plastome genes while the others are nuclear encoded [18]. Protection of the photosynthetic machinery against oxidative stress under excessive light conditions is mediated by the NDH complex [18], [19]. Under abiotic environmental stresses such as low light, drought, high or low temperature, plants involve the NDH complex mediated CET to control the NADPH/ATP ratio and reduced redox intermediates generated from PSII (reviewed in [20]).

The CET around PSI is known to occur via two cyclic electron flow pathways involving Fd-PQ reductase, one the NDH complex [21], [22], [23], [24] and the other a PGR5 (87) and PGR5-Like Photosynthetic Phenotype 1 (PGRL1) as its essential components first reported in Arabidopsis [25], [26]. The NDH complex and PGR5/PGRL1 mediated CEF complement each other due to some redundant components driving the CET [26]. The PGR5/PGRL1-mediated CEF pathway is proposed to be the primary route for electron flow in the ETC around PSI while the NAD complex mediated CEF pathway plays a compensatory role in the CET [26]. Reduced rate of electron transport through PSI with low CO_2_ assimilation under low light intensity in NDH knockout rive mutants indicates the NDH complex significantly contributes to normal growth and yield under low light [27], [28]. The PGR5/PGRL1-dependent CEF pathway is reported to preferentially operates at high light intensities [29].

Most plants contain all the *ndh* genes except for a few plants showing complete or some level of heterotrophy (myco-heterotrophic) such as parasitic plants, submerged plants and some epiphytes. Loss of *ndh* genes is also reported in complete autotrophs such as those within the Gnetales and some conifers belonging to the *Pinaceae* [30], [31], [32], [33], [34], [35], [36], [37]. The possible implications of the missing *ndh* genes to the photosynthetic machinery has been well-reported (reviewed in [38]). For example, heterotrophic parasitic plants such *Cascuta reflexa* and *Epifagus virginiana*, a root holoparasite, lack *ndh* genes and have low or no photosynthetic activities [36], [37]. Several of the *ndh* genes are either lost or pseudogenised in some heterotrophic members of the family Orchidaceae [39], [40]. Impaired photosynthesis was observed in tobacco plants with inactivated *ndh* genes, under fluctuating light intensities, CO_2_ levels or under humidity stress [41], [42].

Here, we report that the plastid genome of jojoba (*Simmondsia chinensis* C.K. Schneid.) has five missing /truncated (pseudogenised) *ndh* genes, with a shortened SSC region and expanded IR regions. Jojoba is well-adapted to grow in hot and dry climates with adequate sunlight and well drained soils. The loss/pseudogenisation of some *ndh* genes, resulting in a defective NDH complex indicates other possible mechanisms negating/offsetting the loss, allowing the survival of jojoba in a harsh growth environment.

## 2 Material and methods

### 2.1 Plant materials and whole genome DNA extraction and sequencing

Complete details of leaf collection, DNA extraction and sequence data and access are mentioned elsewhere [43]. Briefly, male and female jojoba plants were grown from the seeds which were obtained from Saudia Arabia (SA). Young leaf tissue was selected for genomic DNA extraction [44] and subjected to long-read HiFi sequencing using the PacBio Sequel II platform and for short-read (150 bp PE reads) sequencing using the Illumina platform (NovaSeq 6000). Genomic DNA from leaf tissue was extracted also from two other male jojoba varieties (Daddi-Daddi and T100) and two other female jojoba varieties (Wadi-Wadi and Q103), collected randomly from the ‘Chris-Egan’ farm at Inglewood (151°4′.20"E, 28°25'13"S), Queensland, Australia, and subjected to short-read (150 bp PE reads,) sequencing using the Illumina platform (NovaSeq 6000).

### 2.2 Plastid genome assembly using illumina reads

Paired end (PE) Illumina short read (150 bp) data was generated separately from DNA of the SA male jojoba plant and from DNA of the SA female jojoba plant. Plastid genomes (plastomes) of the SA male jojoba and the SA female jojoba were assemble separately using their corresponding Illumina short read datasets. Before plastome sequence assembly, the Illumina PE sequence data sets were quality trimmed using CLC Genomics at 0.01 quality limit (resulting trimmed data with Phred Score over 20 and more than 95% of data with Phred score over 30), which included over 160 million reads as paired end (PE) reads and a total size of 22 Gbp equating to 22X genome coverage. The jojoba plastome was assembled by two approaches to determine any differences in the assembled plastomes. One approach was by *de novo* assembly using the “GetOrganelle” analysis pipeline https://github.com/Kinggerm/GetOrganelle [45]. The other approach [46] using analysis tools available in the CLC genomics Workbench (Qiagen, USA), which involved a combination of “*de novo*-assembly” and “reference plastome sequence guided mapping-assembly”. The plastome of *Chenopodium quinoa* (QuinoaCp): KY419706 [47] was used as the reference in the mapping assembly as it satisfied two criteria, a) like jojoba it lies within the Caryophyllales, the same order which Simmondsia belongs to, and b) has average size of the IR and SSC so as not to bias the mapping derived assembly. Briefly, using CLC-tools, separate plastome sequences generated by the “*de novo* assembly tool” and the “Mapping assembly tool” were aligned to identify mismatches, and if present were manually curated by interrogating corresponding read mapping files for aberrant reads mapped at the mismatch locations. The manual curation corrects for mismatches on either one or both the plastome sequences (“*de novo*-assembly derived” and the “mapping assembly derived” plastomes) to finally generate a “*de novo* and mapping” assembled and curated plastome sequence.

### 2.3 Structural gene annotations of the assembled jojoba plastome

The structure of the assembled SA jojoba male plastome sequences (JmaleCp1 and JmaleCp2) were annotated by combination of approaches. The “GeSeq- Annotation of Organellar Genomes” (GeSeq) annotation pipeline was used to identify a) the structure of the plastome based on the non-repeat sequences (LSC and SSC) and the repeat sequences (IR-A and IR-B) and then b) annotate the protein coding genes and tRNA genes. GeSeq is a freely available online analysis software on CHLOROBOX (https://chlorobox.mpimp-golm.mpg.de/geseq.html). One of the two plastome sequences, the JmaleCp2 referred to as the JojobaCp was compared to the QuinoaCp sequence (*Chenopodium quinoa*, KY419706) using the softwares “Clone Manager” (Sci Ed, USA) and “Geneious” (Biomatters Ltd, USA).

### 2.4 Confirmation of the expanded IR and shortened SSC region: Long PacBio reads

JojobaCp-specific PacBio long read sequences spanning the entire SSC region and part of the bordering IRa and IRb region on either side of the SSC (Part IRa-SSC-Part IRb) were used to confirm the expanded IR region and the shortened SSC region. Jojoba (male from Saudia Arabia) genomic DNA was subjected to PacBio HiFi sequencing. HiFi reads from one SMRT cell (SMRT cell 084320) were processed, and 870,229 CCS reads at quality >Q20 were taken for BLAST analysis (blastn) using the JojobaCp as the target. The first step involved the identification of high confidence JojobaCp-specific PacBio HiFi CCS reads. BLAST analysis (blastn) was undertaken (CLC Genomics Workbench, Qiagen) at the following settings: Expectation: 0.01; Word size: 11; Mask low complexity regions: Yes; Maximum number of hits: 3. Following the blastn analysis, high confidence JojobaCp-specific HiFi CCS reads were identified as follows. Query HiFi CCS reads were first filtered for “Greatest HSP length” at or above 15,000 bp; thereby, identifying 46,590 CCS HiFi reads which were further filtered for “Greatest HSP length” as a percentage of query sequence length at or above 80%, thereby identifying 45,966 as the “high confidence JojobaCp specific PacBio HiFi CCS reads”.

The next step involved identifying high confidence plastome-specific PacBio HiFi CCS reads spanning the SSC region and part of IRa and IRb region on either side of the SSC region (Part IRa-SSC-Part IRb). This analysis was carried out using the “mapping” tool in the “Geneious Prime” software version 2021.2.2 (Geneious, Biomatters, Auckland, New Zealand), at the following settings: Mapper used: Geneious; Sensitivity: Medium sensitivity/Fast; Fine Tuning: None (Fast Read Mapping); Mapping multiple best matches; Randomly. Mapping was undertaken using the 45,966 blastn-derived “High confidence JojobaCp-specific HiFi reads” and the JojobaCp sequence as the reference. Reads mapping to the region covering the Part IRa-SSC-Part IRb were further selected and extracted using the selection tool within Geneious Prime.

### 2.5 Confirmation of the shortened SSC region: Short-read mapping coverage

Short read illumina reads (150 bp paired end reads) were mapped to two plastome sequences as reference sequences; the JojobaCp sequence assembled in this study and to the previously published Jojoba plastome (NC 040935.1, Yao G. et al., 2019) [48], henceforth referred to as the YaoJojobaCp. Illumina reads of the male and female jojoba varieties from Saudia Arabia were mapped to the reference sequences to check the mapping coverage. In addition, Illumina reads of two other male and female genotypes, “Wadi-Wadi”, “T100″, and “Wadi-Wadi”, “Q103″, respectively, were also used for mapping coverages. Illumina reads were mapped to the reference sequences followed by filtering out single reads and retaining paired end mapped reads. The mapping coverage analysis was undertaken using the mapping tool within the CLC Work bench (Qiagen, USA).

### 2.6 Search for the missing plastome *ndh* genes inserted in the jojoba nuclear genome

The GFF file of the annotated jojoba assembled genome sequence [43] was searched for nuclear inserted copies of the *ndhC*, *ndhF, ndhG, ndhI* and *ndhA* missing from the jojoba plastome.

## 3 Results

### 3.1 Plastid genome assembly and structure of male and female jojoba

Two versions of the plastome sequence, labelled arbitrarily by the “Get Organelle” assembler as Cp1 and Cp2 with a flip-flopped SSC region, were assembled for the male and for the female jojoba. All four Cp sequences, two each from the male and the female plant, were of the same length (156,496 bp). Each of the flip-flopped SSC versions from the jojoba male, JmaleCp1 and JmaleCp2, matched perfectly with the corresponding flip-flopped SSC versions from the jojoba female, JfemaleCp2 and JfemaleCp1, respectively. Alignment of the two plastome versions within the male or female differed only at the 1261 bp flip-flopped SSC region (Supplementary Figure 1). As both the plastome sequence versions from the male matched perfectly with one or the other of the two plastome sequence versions from the female jojoba, we chose to use the two plastome versions from the male, identified as JmaleCp1 and JmaleCp2, for gene structure and annotation analysis. Assembled plastomes using the CLC combined approach, from the male and female jojoba plants, matched perfectly to each other and to the corresponding “Get Organelle” plastomes assembled confirming the reliability of the jojoba plastomes we report.

### 3.2 Characterisation of jojoba plastome

**Fig. 1:**
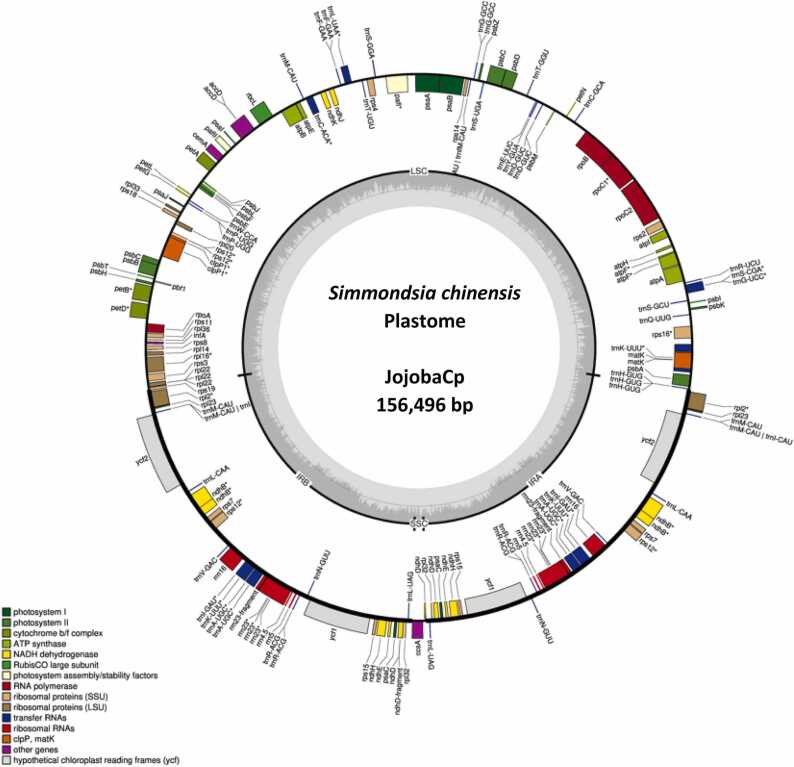
Graphical representation of the annotated jojoba plastome (JojobaCp) sequence. The plastome of jojoba (*Simmondsia chinensis*), JojobaCp sequence (156,496 bp) was assembled using short Illumina sequence reads of a male jojoba plant. Assembly of the plastome was undertaken using “GetOrganelle” analysis pipeline https://github.com/Kinggerm/GetOrganelle.Annotation was undertaken using “GeSeq”, a freely available online analysis software on CHLOROBOX (https://chlorobox.mpimp-golm.mpg.de/geseq.html).

Structural annotation of both the male plastome sequences, JmaleCp1 and JmaleCp2, were the same and consisted of the LSC, IR-A, IR-B and a flip-flopped SSC region (Supplementary Figure 2). The two versions of the male plastome sequences show complete homology, even at the SSC region although inverted. As the JmaleCp2 had the standard structural orientation of >5′LSC3′> >5′IR-A3’>**>5′SSC3′>** <3′IR-B5′< , it was taken for all further analysis and henceforth, throughout the manuscript, referred to as the JojobaCp (Fig. 1).

**Fig. 2:**
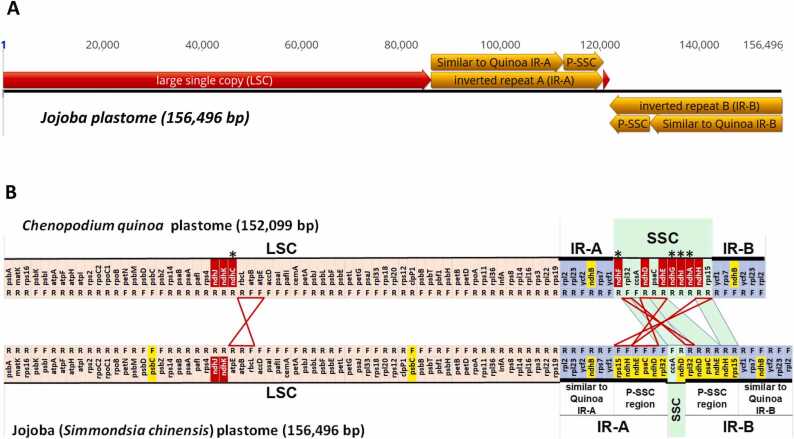
Structural characterisation of the *ndh* genes in the jojoba plastome. A, structural annotation; B, structural and functional annotation of the jojoba plastome sequence; **LCS**, large single copy, **SSC**, small single copy;, **P-SSC**; Part of the SSC region located on the IR region; **IR-A and IR-B**, inverted repeat A and B region respectively; * , *ndh* genes present in the quinoa plastome not present in the jojoba plastome; F, R, genes sequence in same or opposite direction to the plastome sequence orientation 5′->LSC/IR-A/SSC/IR-B-> 3′. Genes located on the LSC, IR and SSC regions are indicated as “cream”, “blue” and “green” highlight respectively. All *ndh* genes when present as a single copy are indicated in “red” highlight. Genes including *ndh* genes when present as multiple copies are indicated in “yellow” highlight. Presence of *ndh* genes in both quinoa and jojoba plastomes and in the same direction are indicated as “green bars with blue borders” and in the reverse direction are indicated as red bordered hourglass symbol. Five of the eleven *ndh* genes*, ndhC, ndhF, ndhG, ndhI* and *ndhA*, are missing in the jojoba plastome. Sequence maps in A and B are not at the same length scale.

Alignment of the JojobaCp IR sequence (34,620 bp) to the quinoa plastome (QuinoaCp) IR (25,205 bp) revealed a larger JojobaCp IR sequence (34,620 bp) which consists of two parts; a 26,613 bp region with high sequence similarity to the entire QuinoaCp IR region followed by an additional region of 8007 bp (Supplementary Figure 3 A-C). Characterisation of the additional IR-A and IR-B regions, when compared to the QuinoaCp, indicates some of the genes located on the quinoa SSC region are missing from the JojobaCp SSC region but instead are present on the additional IR region of 8007 bp (Supplementary Figure 4). Hence, the IR region in jojoba plastome has expanded to cover part of the SSC region with consequent reduction of the SSC region. The additional region on jojoba IR-A and IR-B containing part of the SSC genes is labelled as “Part-SSC” (P-SSC) (Fig. 2A). As the IR-A and IR-B are inverted copies of each other, so are the P-SSC sequences. The direction of genes of the P-SSC of jojoba IR-B is the same as that found on the quinoa SSC region but is in the opposite direction on the P-SSC of IR-A region.

### 3.3 Jojoba plastome *ndh* gene loss

Three *ndh* genes, *ndhH, ndhE* and *ndhD,* are in the P-SSC regions and thus are present as two copies. The *ndhD* gene is also present on the SSC region and is the only *ndh* gene in this region. As found in the quinoa plastome, the *ndhB* gene is present as a single copy in each of the two IR regions. Of the eleven *ndh* genes generally present in plastomes, five are missing from the jojoba plastome. The *ndhC* is missing from the LSC region and the other four, the *ndhF, ndhG, ndhI* and *ndhA*, are missing from the SSC region (Fig. 2b, Table 1). In addition, the presence of several premature stop codons in the *ndhD* gene indicates the lack of a full-length protein being synthesised. Unlike the QuinoaCp SSC region (18,107 bp) which consists of seven *ndh* genes, the JojobaCp SSC region is only 1261 bp and consists of only the *ndhD* gene with four *ndh* genes missing and three of the *ndh* genes now part of the P-SSC region of the IR. Aligning the QuinoaCp SSC, the JojobaCp SSC and the JojobaCp P-SSC region identified three regions of the QuinoaCp SSC region missing in the JojobaCp SSC region (Supplementary Figure 5). The mechanism leading to the two SSC flip-flop versions of the plastome sequence [49], possibly contributes to the perturbed length of the SSC region where genes are either lost or are part of the IR region.

**Table 1:** Coding sequences on the IR, P-SSC and SSC of jojoba and *Chenopodium quinoa* plastomes.

CDS name	Species	Number of copies	Direction	Location	Nucleotide sequence Length	Amino Acid sequence length	Start ATG present	Premature stop codons
*ycf1*	*C. quinoa*	2	forward	*IR-A & SSC	1554	518	Yes	no
			reverse	*SSC & IR-B	5610	1870	Yes	no
	Jojoba	2	forward	*IR-A & P-SSC	5682	1894	Yes	no
			reverse	*P-SSC & IR-B	5682	1894	Yes	no
*ndhF*	*C. quinoa*	1	reverse	*IR-A & SSC	2247	749	Yes	no
	Jojoba	**Missing**						
*rpl32*	*C. quinoa*	1	forward	SSC	174	58	Yes	no
	Jojoba	2	forward	P-SSC of IR-A	129	43	Yes	no
			reverse	P-SSC of IR-B	129	43	Yes	no
*ccsA*	*C. quinoa*	1	forward	SSC	972	324	Yes	no
	Jojoba	1	forward	SSC	972	324	Yes	no
*ndhD*	*C. quinoa*	1	reverse	SSC	1503	501	no	no
	Jojoba	3	forward	P-SSC of IR-A	448	149	Yes	Yes, several
			reverse	SSC	148	49	no	Yes, several
			reverse	P-SSC of IR-B	448	149	Yes	Yes, several
*psaC*	*C. quinoa*	1	reverse	SSC	246	82	Yes	no
	Jojoba	2	forward	P-SSC of IR-A	246	82	Yes	no
			reverse	P-SSC of IR-B	246	82	Yes	no
*ndhE*	*C. quinoa*	1	reverse	SSC	306	102	Yes	no
	Jojoba	2	forward	P-SSC of IR-A	237	79	no	no
			reverse	P-SSC of IR-B	237	79	no	no
*ndhG*	*C. quinoa*	1	reverse	SSC	531	177	Yes	no
	Jojoba	**Missing**						
*ndhI*	*C. quinoa*	1	reverse	SSC	504	168	Yes	no
	Jojoba	**Missing**						
*ndhA*	*C. quinoa*	1	reverse	SSC	1086	362	Yes	no
	Jojoba	**Missing**						
*ndhH*	*C. quinoa*	1	reverse	SSC	1182	394	Yes	no
	Jojoba	2	forward	P-SSC of IR-A	768	256	Yes	no
			reverse	P-SSC of IR-B	768	256	Yes	no
*rps15*	*C. quinoa*	1	reverse	SSC	273	91	Yes	no
	Jojoba	2	forward	P-SSC of IR-A	273	91	Yes	no
			reverse	P-SSC of IR-B	273	91	Yes	no

SSC, Small single copy; * , CDS spans across two regions as indicated; IR-A & IR-B Inverted Repeat A and B respectively: P-SSC, Part of SSC region now part of the IR region.

### 3.4 Confirmation of plastid genome using long and highly accurate HiFi reads

**Fig. 3:**
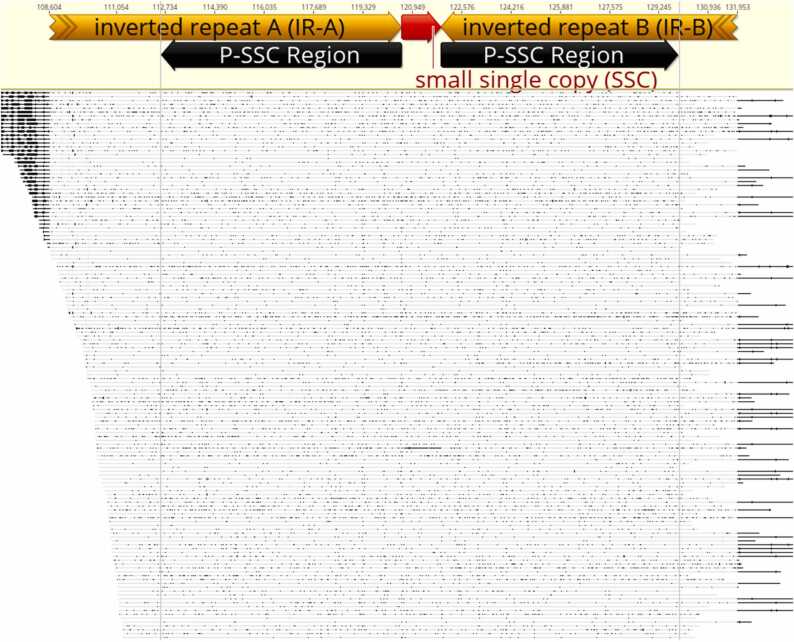
Mapped PacBio HiFi reads spanning the P-SSC of IR-A, SSC and P-SSC of IR-B confirming the expanded IR and reduced SSC regions in the jojoba plastome. IR-A, IR-B, Inverted repeat -A and -B region respectively; SSC, small single copy; P-SSC-Region, part of the SSC region now on the IR region; Cp, Plastome. The mapped PacBio HiFi reads spans the P-SSC of IR-A, SSC and the P-SSC of IR-B sequence.

The expanded IR regions with the orientation of the P-SSC region and the shortened SSC region identified in the JojobaCp genome sequence assembled from short Illumina reads was confirmed using whole genome PacBio HiFi reads of the same jojoba male accession. Blastn selected 45,966 PacBio HiFi reads when aligned to the JojobaCp generated a perfectly matching consensus plastome sequence. Of the 45,966 PacBio HiFi reads, 214 were found to be spanning the P-SSC region of the IR-A, the SSC region and the P-SSC region of the IR-B (Fig. 3**,** Supplementary Data **file 1**) confirming the expanded IR region and shortened SSC region in the JojobaCp sequence we report here. The shortened SSC in the JojobaCp we report here was also confirmed by mapping Illumina reads to the JojobaCp we assembled and the YaoJojobaCp which is another published [48] jojoba plastome sequence with some *ndh* gene loss and having a longer SSC region (Supplementary Figure 6). The Illumina reads of the male Jojoba mapped evenly through the JojobaCp we report but not to the YaoJojobaCp sequence where the mapping coverage drastically reduced in one part of the SSC region (Supplementary Figure 7A and B). Similar results were obtained when whole genome illumina reads from one additional jojoba male and two jojoba female accessions were mapped to the JojobaCp and the YaoJojobaCp sequence (Supplementary Figure 8).

### 3.5 Truncated nuclear copy of the *ndhF* gene

Of the five-missing jojoba plastome *ndh* genes (*ndhA, ndhC, ndhI, ndhG, ndhF),* a truncated sequence of the plastome *ndhF* gene was found in the jojoba nuclear genome. The nuclear sequence is much smaller (375 bp) when compared to the quinoa plastome *ndhF* gene (2247 bp) but shows 34% pairwise sequence identity (Supplementary Figure 9).

## 4 Discussion

We assembled jojoba plastomes with GetOrganelle, two versions each for the SA jojoba male and for the SA jojoba female. Both versions as expected [50], [51] were identical except for the flip-floppd SSC region (>5′LSC3′>>5′IR-A3’>**>5′SSC3′>**<3′IR-B5′< and >5′LSC3′>>5′IR-A3′>**<3′SSC5**’**<**<3′IR-B5′<). One of the two plastomes from the SA male jojoba, the JojobaCp, with the standard orientation (>5′LSC3′>>5′IR-A3’>**>5′SSC3′>**<3′IR-B5′<), was taken for all further analysis. Although the JojobaCp sequence has the usual plastome structure, the length of the IR and SSC is not what is typically found in plastomes of land plants [13], [52]. In angiosperms, the plastome size ranges from 120 kb to 160 kb and is depicted as a quadripartite structure consisting of a large single copy (LSC) region (average size 80 kb) and a small single copy (SSC) region (average size 20 kb), separated by two inverted repeat (IR-A and IR-B) regions (each of average size 25 kb) [52], [53].

Comparison of the jojoba plastome sequence to that of quinoa, revealed the length of the SSC in the JojobaCp (1261 bp) to be much smaller than the SSC in the QuinoaCp (18,107 bp). Whole genome PacBio HiFi reads mapped across the SSC region of the JojobaCp including boarders of the IR-A and IR-B regions confirmed the shortened SSC region (Fig. 3, Supplementary Data **file 1**). In addition, the length of the JojobaCp IR region (34,620 bp) is longer than the QuinoaCp IR region (25,205 bp) (Table 2). The size of the IR region ranges from 20 to 30 kb in most angiosperms while in non-seed plants ranges from 10 to 15 kb [13], (reviewed in [20]).

**Table 2:** Comparison of plastome sequence structures in Jojoba and other selected species.

Plastid annotations were obtained from published data or by GeSeq derived annotations. Green and brown cells, polycistronic and monocistronic genes; number and location of genes located on LSC or IR or SSC region is indicated as red or yellow or blue cells respectively; grey cells, no structural annotations by GeSeq due to absence of two inverted repeats; * , gene present with truncation or frameshift; OP, obligate parasitic, PA, parasitic; MA, mycorrhizal association; LSC, Large Single Copy; IR-A and IR-B, Inverted Repeat -A and -B respectively; SSC, Small Single Copy; CLC, CLC Genomics Workbench (Qiagen, USA); GetOrganelle, https://github.com/Kinggerm/GetOrganelle; Geseq, https://chlorobox.mpimp-golm.mpg.de/geseq.html.

A shortened jojoba plastome SSC region has resulted in the loss of four *ndh* genes *ndhF*, *ndhG, ndhI*, *ndhA.* Plastomes have lost thousands of genes from the original cyanobacteria from which they have evolved, with some genes integrated in the nucleus [54]. Although the huge loss of plastome genes to the nucleus renders the plastids semiautonomous, between 20 and 200 genes are involved in the photo-metabolic process including photosynthesis are maintained and retained in plastids, including eleven *ndh* genes [7]. In most angiosperms the structure of the plastome sequence and the gene content and order are remarkably conserved [13], with a majority of these arranged in operons and transcribed as polycistronic precursor molecules which are then cleaved to generate the mature mRNA [33], [55], [56], [57]. The eleven *ndh* genes (*ndh*A, to *ndhK*) from the plastids, along with more than 19 nuclear genes, encode subunits of the NADH-dehydrogenase (NDH) complex [17]. In plastomes, most of the 11 *ndh* genes are arranged as operons, and their polycistronic transcription in jojoba plastome may be perturbed due to the loss of five *ndh* genes. Loss of three *ndh* genes, *ndhG, ndhI* and *ndhA* in the JojobaCp due to a shortened SSC region may affect expression of the remaining subunits of the *ndhH-D* operon comprising of the *ndhH*, *ndhA*, *ndhI*, *ndhG, ndhE* and the *ndh*D genes [58]. Likewise, loss of the *ndh*C from the LSC region in the JojobaCp may perturb the expression of the *nhdK* and the *nhdJ* subunits of the *ndh*C-J operon (*ndh*C, *ndh*K and *ndh*J genes) [59]. In addition, the *ndhF* is also lost from the SSC region in the JojobaCp. Of the five missing plastome *ndh* genes, a truncated copy (375 bp) of only the *ndhF* was identified in the nuclear genome which is smaller than the plastome *ndhF* gene (2247 bp) (Supplementary Figure 9**)**. The loss of five of the eleven *ndh* genes in JojobaCp can lead to a defective NDH complex.

The main role of chloroplasts, a type of plastome, is to carry out photosynthesis along with other central roles involving the synthesis of amino acids, starch, fatty acids and pigments [60]. The NDH complex plays a crucial role in photosynthesis via the two photosynthetic rection centres, Photosystem II (PSII) and Photosystem I (PSI). In plant chloroplasts, light is harvested by PSII and PSI, and the generated excited electrons are transported via a chain of several redox intermediates known as the electron transport chain (ETC). The ETC is further defined as a linear electron transport (LET) chain or a cyclic electron transport chain (CET) depending on a linear flow (LEF) or cyclic flow (CEF), respectively, of electrons via the redox intermediates. The LET involves electron flow via the PSII and PSI redox intermediates to generate ATP and NADPH. The CET involves electron flow via the PSI redox intermediates to generate ATP [61], [62]. The NDH complex is involved in the recycling of electrons around PSI via the CET, essentially acting as a valve to poise the redox levels (reduced/oxidised ratio) of the ETC intermediaries. Therefore, the NDH complex plays a crucial role to optimise the rate of the ETC under various situations [62]. For example, electron supply from PSII is generally inadequate to generate the required amount of ATP for the CO_2_ assimilation via the Calvin cycle [63], (reviewed in [20], [64]). Under this situation, the NDH complex mediates the flow of electrons from ferredoxin (Fd), away from steps for NADPH generation but instead through the CET chain leading to the generation of ATP. In the CET chain, the electrons from ferredoxin (Fd) are recycled back to plastoquinone (PQ), to Cyt *b*_*6*_*f*, plastocyanin (Pc) and PSI reaction centre (P_700_) to generate ATP (reviewed in [15]).

Another role of the NDH complex is to protect plants against photodamage of the photosystems [65], [66] caused by stress from strong light, high heat or low temperature [67], [68], [69], [70], [71]. Photodamage of PSII and PSI is caused when there is a build-up of reductants (substrates and products) in the LET and of excited electrons causing membrane oxidation from reactive oxygen species [72], [73]. During the first stages of photooxidative stress due to sudden increase in light intensity, the NDH complex has been linked to the draining of excess excited electrons via the Mehler reaction [74], [75] and the generation of reactive oxygen species (ROS) which has been directly linked to tissue damage [76]. The concerted action of scavenging enzymes superoxide dismutase (SOD) [62], [77], [78] and plastoquinol terminal oxidase / plastid terminal oxidase (PTOX) [71], [79], [80] reduces this damage. However, in mature leaves the activity of the NDH is increased [62], [81], [82], [83], [84] and SOD is reduced leading to programmed cell death (PCD) [85], [86], [87]. Conversely, the supply of electrons from PSII is temporarily reduced under conditions of rapid decrease in light intensity or during early stages of plastome biogenesis, [77], [82] or in the first few minutes post photoinhibition of PSII [88]. Under these conditions the NDH complex along with chlororespiration also acts to poise the redox level of the ETC intermediaries; thus, playing a role in protection against photooxidative stress [62]. It is intriguing, how jojoba as an autotrophic plant is able to avoid abiotic oxidative stress with loss of five of the eleven *ndh* genes and possibly a defective NDH complex.

Several plants have been reported to have lost some or all the plastid *ndh* genes, a loss not related to the loss of genes as it evolved from the captured cyanobacteria. Instead, the *ndh* gene loss in plant plastids can be attributed to environmental reasons in some but not in all cases [38]. With some exceptions, the entire set of the eleven *ndh* genes are present in bryophytes, ferns and in photosynthetic higher plants (Table 2). Loss or pseudogenisation of the *ndh* genes in Angiosperms and Gymnosperms is not consistent in all species within a genera (reviewed in [38]). Some of the *ndh* genes are lost or pseudogenized in parasitic plant lineages, heterotrophic species of family Orchidaceae, cactaceae and some submerged plants including in Gnetales and in conifers (mainly Pinaceae) [30], [35], [36], [37], [39], [77], [89], [90].

Plants surviving without *ndh* genes have evolved with morphological adaptations for complete heterotrophy as in parasitic plants or mycoheterotrophy as in *Epifagus* [34], some orchid species [40] and Cuscuta [35], [36]. Heterotrophy in parasitic plants, would have evolved with successive events of *ndh* gene loss and of additional plastome genes involved in the photosynthetic apparatus. However, loss of the *ndh* genes would have also involved the co-evolving from transitory to complete parasitism and in some cases with morphological changes such as haustoria development to aid in accessing nutrients from the host plants [34], [35], [36], [37], [91], [92]. Epifagus is a root parasite and considered a holoparasite [34]. *Aneura mirabilis,* a parasitic liverwort, exhibits mycoheterotrophy or epiparasitism with a completely non photosynthetic life history due to its mycorrhizal association with a basidiomycete fungus which in turn extracts nutrients from a host tree [93]. The loss of the plastid encoded NDH subunit genes in these plants having some level of heterotrophy indicates the *ndh* genes to be dispensable. However, without functional or morphological adaptations in place or co-evolved in parallel with loss of the *ndh* genes, these plants would be at risk of survival under abiotic stress conditions and eventually lead to extinction [38].

Autotrophic angiosperms with lost *ndh* genes are rare. Within the family Orchidaceae, evolutionary analysis of plastid genomes of species with all or some of the *ndh* genes (ndh-complete and ndh-deleted type), indicates a compensatory evolutionary transition from phototrophic to myco-heterotrophic or fully heterotrophic metabolism facilitated with suitable structural adaptations [20], [30], [39], [90], [94]. Other plants without *ndh* genes exist as they have functional adaptations to continue being autotrophic, such as crassulacean acid metabolism (CAM) in cactus [95] or in sea grass which evolved from terrestrial monocots to adapt and flourish in the transition zone between the sea and the terrestrial land environment. Studies on the sea grass species *Zostera marina and Zostera muelleri,* have revealed adaptive mechanisms that led to the loss of certain nuclear genes commonly present in terrestrial plants. These genes include those controlling stomatal opening and cell wall metabolism. However, they also gained crucial nuclear genes responsible for controlling gas exchange and nutrient absorption, which are essential for their adaptation to the complex marine environment characterized by high salt levels, high osmotic pressure, and low light conditions [96], [97], [98], [99].

The evolutionary pressure on the *ndh* genes between requirement or dispensability challenged by the lack of functional advantages under less harsher environments, may have led some plants to lose these genes but with a reduced adaptive ability to cope with environmental change. Mutants lacking NDH complex exhibit growth defects in response to different stress conditions, including high light, water deficiency and low temperature (reviewed in [100]). Transgenic plants with a defective *ndh* gene and hence a defective NDH complex, grow normally under mild environmental conditions but not under stress conditions and have an impaired photosynthetic rate under rapidly fluctuating light intensities [77]. Loss of the *ndh* genes by plants under milder environments rendered them unable to survive in harsher or stress environments leading to the endpoint of their evolution [38]. Positive selection of the *ndh* genes in species within the genus Allium is suggested as a mechanism to cope with the constant excessive light as against *A. paradoxum* which lost or pseudogenised all its *ndh* genes and grows in shady humid forests [101]. Similar observations in the disparity of the loss of *ndh* genes and adaptations was reported in the semi-aquatic *Saniculiphyllum guangxiense* an endangered species, which is in contrast to the conservation of the *ndh* genes in the other Saxifragales [102]. *Haberlea rhodopensis*, the only member within the genus, is a member of the resurrection plant group. It retains all the plastome *ndh* genes, with positive selection detected at specific sites within the *ndhF* gene, possibly influencing its translational control. This adaptation enables the species to cope with repeated desiccation and rehydration events by delaying senescence. Retention of all *ndh* genes with positive selection of specific sites within the *ndhF* gene, probably affecting its translational control, allows *Haberlea rhodopensis*, a resurrection species, to cope with repeated desiccation and rehydration by delaying senescence [103]. Delayed senescence was observed in transgenic tobacco with an *ndh*F knock-out [104]. The impact on senescence in jojoba due to the missing *ndhF* gene, if any, needs to be elucidated.

The dispensability of the *ndh* genes in some members of the Orchidaceae [30] and in the gymnosperms (some lines of the Gnetales and conifers) which are fully photosynthetic is not clearly understood [105]. One reason as suggested by others T Shikanai [18], [106] for the loss of the *ndh* genes in conifers may be due to less photooxidative stress associated with high atmospheric CO_2_ during the carboniferous period [107]. This suggestion may hold some credibility as transgenic tobacco plants with a defective *ndhB* gene exhibited impaired photosynthetic rates at ambient CO_2_ levels coupled with humidity stress with partial stomatal closure but not under increased CO_2_ concentrations [42].

Throughout evolution, land plants which lost their *ndh* genes may have become extinct under challenging climactic conditions. Those plants without *ndh* genes existing today would have evolved from a more recent event (less than 10 Mya) as suggested by Sabater B. 2021 [38]. Plants without *ndh* genes may have become extinct progressively if faced with stressful climate conditions unless they develop functional protective pathways or morphological adaptions to transition from autotrophy to heterotrophy [38]. Loss of *ndh* genes in *Kingdonia uniflora* [108] renders it critically endangered. Similarly, the loss of the *ndh*F gene in *Mikania cordata* restricts it to a stable and less stressful habitat contrasting to the more invasive habitat of *Mikania micrantha* which retains all the *ndh* genes [109].

It is possible for the PGR5/PGRL1-dependent CET to preferentially operate in jojoba in the generation of ATP. Under high light, PSII is reported to be more sensitive than PSI [110] and the PGR5/PGRL1-dependent CEF pathway in jojoba may also contribute to the photoprotection of both PSII and PSI as is the case in Arabidopsis [26]. With high light and high temperature growth habitat of jojoba, there could be two mechanisms in place to protect from photo inhibition/photodamage. One is the efficient dissipation of excess photon energy as heat via the non-photochemical quenching (NPQ) of chlorophyll fluorescence, which in Arabidopsis is mediated by the PsbS and Xanthin cycle. The other, the PGR5/PGRL1-dependent CET [26], [111], [112], where in Arabidopsis, the PGR5 downregulates the Cyt *b*_*6*_*f* complex [113]. This process ensures the photoprotection of the PSI and PSII due to the reduction of the flow of excited electrons via PSII and PSI coupled with the draining of excess electrons as reduced PQ pool via the Mehler reaction. Excessive generated ROS can cause tissue damage if not scavenged by SOD [62], [77], [78] and PQTO [71], [79], [80] [76]. The measurement of SOD and PQTO in jojoba may shed light on how it manages ROS without membrane damage. It is possible that the partial stomatal closure due to excessive heat and waster stress may contribute to reduced CO_2_ fixation and slow growth, although investigations are required into established mechanisms of increasing ATP/NADPH ratio via the LET, CET mediated by PGR5/PGRL1, and the Mehler reactions.

The fact that jojoba, the only species within its genus *Simmondsia*, has some missing *ndh* genes suggests that other members of these species might be extinct due to their inability to adapt to a harsher environment. However, jojoba may have survived due to reasons including the presence of an intact photosynthesis machinery, like the PGR5/PGRL-1 pathway contributing to the CEF generating the required ATP and having alternate stress response mechanisms to protect the plant. Like jojoba, *Welwitschia mirabilis* the only species in the Gnetales, also lacks the *ndh* genes but is known to live up to 1000 years [114].

Survival and longevity of Gnetales and some species in *Pinus* can be attributed to the loss of the *ndh* genes, especially the *ndhF* gene. Delayed senescence was observed in transgenic tobacco with an *ndh*F knock-out [104]. Jojoba, native and adapted to grow in a harsh environment of the tropical and warm temperate desert regions of the southwestern United States and northern Mexico[115], [116], is a slow growing evergreen shrub having a live span between 100 and 200 years [117]. Interestingly, jojoba lacks five of the 11 *ndh* genes including the *ndhF* gene which could contribute to delayed senescence and its longevity.

## 5 Conclusions

Several studies suggest the loss of *ndh* genes leading to a defective NDH complex in plants is dispensable under mild and non-stressing environmental habitats. What is unclear is how gymnosperms are protected by abiotic stress without the *ndh* genes, when angiosperms are thought to be protected with these genes. Also, not clear is how jojoba, an Angiosperm growing in harsh environmental habitats with severe drought and cold stress, survive without all of the 11 *ndh* genes and yet maintain photosynthesis. What is clear though in jojoba is that the loss of five *ndh* genes leading to a defective NDH is inconsequential to the survival of jojoba even in harsh environments escaping extinction without evolving into a heterotroph via a parasitic or carnivorous nature to compensate for low photosynthetic rate.

## CRediT authorship contribution statement

RH, IA-M, AK, AF: Conceptualization; OA, BA, AK, AF: Data curation; RH, IA-M, AK, AF, AW: Formal analysis; RH, IA-M, AK: Funding acquisition; RH, IA-M, AK, AF: Investigation; RH, AK, AF: Methodology; RH, AK, AF: Project administration; RH, IA-M, AK: Resources; AK, AF: Software; RH, IA-M, AK, AF: Supervision; AK, AF: Validation; AK, AF: Visualization; AK, AF: Roles/Writing - original draft; All authors: Writing - review and editing.

## Declaration of Competing Interest

The authors declare no competing interests.

## Data Availability

All sequence data can be accessed at NCBI via BioProject# PRJNA912913. Short read Illumina datasets can be accessed via BioSample #s SAMN32262371_ SAMN32262372_ SAMN32262373_ SAMN32262374_ SAMN32262375_ SAMN32262376. PacBio HiFi (CCS) data can be accessed via BioSample #s SAMN32299510 and SAMN32299511.

## References

[1] He H., Wu X., Xian H., Zhu J., Yang Y., Lv Y. (2021). An abiotic source of Archean hydrogen peroxide and oxygen that pre-dates oxygenic photosynthesis. Nat Commun.

[2] Farquhar J., Zerkle A.L., Bekker A. (2011). Geological constraints on the origin of oxygenic photosynthesis. Photosynth Res.

[3] Holland H.D. (2006). The oxygenation of the atmosphere and oceans. Philos Trans R Soc Lond Ser B, Biol Sci.

[4] Bhattacharya D., Yoon H.S., Hackett J.D. (2004). Photosynthetic eukaryotes unite: endosymbiosis connects the dots. Bioessays.

[5] Yoon H.S., Hackett J.D., Ciniglia C., Pinto G., Bhattacharya D. (2004). A molecular timeline for the origin of photosynthetic eukaryotes. Mol Biol Evol.

[6] Butterfield N.J. (2000). Bangiomorpha pubescens n. gen., n. sp.: implications for the evolution of sex, multicellularity, and the mesoproterozoic/neoproterozoic radiation of eukaryotes. Paleobiology.

[7] Martin W. (2003). Gene transfer from organelles to the nucleus: frequent and in big chunks. Proc Natl Acad Sci USA.

[8] Martin W., Rujan T., Richly E., Hansen A., Cornelsen S., Lins T. (2002). Evolutionary analysis of Arabidopsis, cyanobacterial, and chloroplast genomes reveals plastid phylogeny and thousands of cyanobacterial genes in the nucleus. Proc Natl Acad Sci USA.

[9] Archibald J.M. (2011). Origin of eukaryotic cells: 40 years on. Symbiosis.

[10] Green B.R. (2011). Chloroplast genomes of photosynthetic eukaryotes. Plant J.

[11] Blankenship R.E. (2021). Molecular Mechanisms of Photosynthesis.

[12] Martin T., Oswald O., Graham I.A. (2002). Arabidopsis Seedling Growth, Storage Lipid Mobilization, and Photosynthetic Gene Expression Are Regulated by Carbon:Nitrogen Availability. Plant Physiol.

[13] Wicke S., Schneeweiss G.M., dePamphilis C.W., Müller K.F., Quandt D. (2011). The evolution of the plastid chromosome in land plants: gene content, gene order, gene function. Plant Mol Biol.

[14] Daniell H., Lin C.-S., Yu M., Chang W.-J. (2016). Chloroplast genomes: diversity, evolution, and applications in genetic engineering. Genome Biol.

[15] Krieger-Liszkay A., Kirilovsky D., Ruban A., Foyer C.H., Murchie E.H. (2022). *Chapter 2 - Transport of electrons*, in *Photosynthesis in Action*.

[16] Raines C.A., Cavanagh A.P., Simkin A.J., Ruban A., Foyer C.H., Murchie E.H. (2022). *Chapter 9 - Improving carbon fixation*, in *Photosynthesis in Action*.

[17] Ifuku K., Endo T., Shikanai T., Aro E.M. (2011). Structure of the chloroplast NADH dehydrogenase-like complex: nomenclature for nuclear-encoded subunits. Plant Cell Physiol.

[18] Shikanai T. (2016). Chloroplast NDH: a different enzyme with a structure similar to that of respiratory NADH dehydrogenase. Biochim Biophys Acta - Bioenerg.

[19] Endo T., Shikanai T., Takabayashi A., Asada K., Sato F. (1999). The role of chloroplastic NAD(P)H dehydrogenase in photoprotection. FEBS Lett.

[20] Ma M., Liu Y., Bai C., Yong J.W.H. (2021). The Significance of Chloroplast NAD(P)H Dehydrogenase Complex and Its Dependent Cyclic Electron Transport in Photosynthesis. Front Plant Sci.

[21] Joët T., Cournac L., Horvath E.M., Medgyesy P., Peltier G. (2001). Increased sensitivity of photosynthesis to antimycin A induced by inactivation of the chloroplast ndhB gene. Evidence for a participation of the NADH-dehydrogenase complex to cyclic electron flow around photosystem I. Plant Physiol.

[22] Hashimoto M., Endo T., Peltier G., Tasaka M., Shikanai T. (2003). A nucleus-encoded factor, CRR2, is essential for the expression of chloroplast ndhB in Arabidopsis. Plant J.

[23] Shikanai T., Endo T., Hashimoto T., Yamada Y., Asada K., Yokota A. (1998). Directed disruption of the tobacco ndhB gene impairs cyclic electron flow around photosystem I. Proc Natl Acad Sci USA.

[24] Burrows P.A., Sazanov L.A., Svab Z., Maliga P., Nixon P.J. (1998). Identification of a functional respiratory complex in chloroplasts through analysis of tobacco mutants containing disrupted plastid ndh genes. EMBO J.

[25] DalCorso G., Pesaresi P., Masiero S., Aseeva E., Schünemann D., Finazzi G. (2008). A complex containing PGRL1 and PGR5 is involved in the switch between linear and cyclic electron flow in Arabidopsis. Cell.

[26] Munekage Y., Hojo M., Meurer J., Endo T., Tasaka M., Shikanai T. (2002). PGR5 is involved in cyclic electron flow around photosystem I and is essential for photoprotection in Arabidopsis. Cell.

[27] Yamori W., Shikanai T., Makino A. (2015). Photosystem I cyclic electron flow via chloroplast NADH dehydrogenase-like complex performs a physiological role for photosynthesis at low light. Sci Rep.

[28] Ueda M., Kuniyoshi T., Yamamoto H., Sugimoto K., Ishizaki K., Kohchi T. (2012). Composition and physiological function of the chloroplast NADH dehydrogenase-like complex in Marchantia polymorpha. Plant J.

[29] Wang C., Yamamoto H., Shikanai T. (2015). Role of cyclic electron transport around photosystem I in regulating proton motive force. Biochim Biophys Acta - Bioenerg.

[30] Lin C.-S., Chen J.J.W., Huang Y.-T., Chan M.-T., Daniell H., Chang W.-J. (2015). The location and translocation of ndh genes of chloroplast origin in the Orchidaceae family. Sci Rep.

[31] Köhler M., Reginato M., Souza-Chies T.T., Majure L.C. (2020). Insights into chloroplast genome evolution across opuntioideae (Cactaceae) reveals robust yet sometimes conflicting phylogenetic topologies. Front Plant Sci.

[32] Braukmann T.W., Kuzmina M., Stefanović S. (2009). Loss of all plastid ndh genes in Gnetales and conifers: extent and evolutionary significance for the seed plant phylogeny. Curr Genet.

[33] Wakasugi T., Tsudzuki J., Ito S., Nakashima K., Tsudzuki T., Sugiura M. (1994). Loss of all ndh genes as determined by sequencing the entire chloroplast genome of the black pine Pinus thunbergii. Proc Natl Acad Sci USA.

[34] dePamphilis C.W., Palmer J.D. (1990). Loss of photosynthetic and chlororespiratory genes from the plastid genome of a parasitic flowering plant. Nature.

[35] McNeal J.R., Kuehl J.V., Boore J.L., de Pamphilis C.W. (2007). Complete plastid genome sequences suggest strong selection for retention of photosynthetic genes in the parasitic plant genus Cuscuta. BMC Plant Biol.

[36] Funk H.T., Berg S., Krupinska K., Maier U.G., Krause K. (2007). Complete DNA sequences of the plastid genomes of two parasitic flowering plant species, Cuscuta reflexa and Cuscuta gronovii. BMC Plant Biol.

[37] Wolfe K.H., Morden C.W., Palmer J.D. (1992). Function and evolution of a minimal plastid genome from a nonphotosynthetic parasitic plant. Proc Natl Acad Sci USA.

[38] Sabater B. (2021). On the edge of dispensability, the chloroplast ndh genes. Int J Mol Sci.

[39] Barrett C.F., Freudenstein J.V., Li J., Mayfield-Jones D.R., Perez L., Pires J.C. (2014). Investigating the path of plastid genome degradation in an early-transitional clade of heterotrophic orchids, and implications for heterotrophic angiosperms. Mol Biol Evol.

[40] Kim H.T., Kim J.S., Moore M.J., Neubig K.M., Williams N.H., Whitten W.M. (2015). Seven new complete plastome sequences reveal rampant independent loss of the ndh gene family across orchids and associated instability of the inverted repeat/small single-copy region boundaries. PLOS ONE.

[41] Martín M., Noarbe D.M., Serrot P.H., Sabater B. (2015). The rise of the photosynthetic rate when light intensity increases is delayed in ndh gene-defective tobacco at high but not at low CO_2_ concentrations. Front Plant Sci.

[42] Horváth E.M., Peter S.O., Joët T., Rumeau D., Cournac L., Horváth G.V. (2000). Targeted inactivation of the plastid ndhB gene in tobacco results in an enhanced sensitivity of photosynthesis to moderate stomatal closure. Plant Physiol.

[43] Al-Dossary O., Alsubaie B., Kharabian-Masouleh A., Al-Mssallem I., Furtado A., Henry R.J. (2021). The jojoba genome reveals wide divergence of the sex chromosomes in a dioecious plant. Plant J.

[44] Furtado A., Henry R.J., Furtado A. (2014). DNA Extraction from Vegetative Tissue for Next-Generation Sequencing, in *Cereal Genomics*.

[45] Jin J.-J., Yu W.-B., Yang J.-B., Song Y., dePamphilis C.W., Yi T.-S. (2020). GetOrganelle: a fast and versatile toolkit for accurate de novo assembly of organelle genomes. Genome Biol.

[46] Moner A.M., Furtado A., Henry R.J. (2018). Chloroplast phylogeography of AA genome rice species. Mol Phylogen Evol.

[47] Hong S.-Y., Cheon K.-S., Yoo K.-O., Lee H.-O., Cho K.-S., Suh J.-T. (2017). Complete chloroplast genome sequences and comparative analysis of Chenopodium quinoa and C. album. Front Plant Sci.

[48] Yao G., Jin J.J., Li H.T., Yang J.B., Mandala V.S., Croley M. (2019). Plastid phylogenomic insights into the evolution of Caryophyllales. Mol Phylogenet Evol.

[49] Oldenburg D.J., Bendich A.J. (2004). Most chloroplast DNA of maize seedlings in linear molecules with defined ends and branched forms. J Mol Biol.

[50] Wang W., Lanfear R. (2019). Long-reads reveal that the chloroplast genome exists in two distinct versions in most plants. Genome Biol Evol.

[51] Palmer J.D. (1983). Chloroplast DNA exists in two orientations. Nature.

[52] Xiao-Ming Z., Junrui W., Li F., Sha L., Hongbo P., Lan Q. (2017). Inferring the evolutionary mechanism of the chloroplast genome size by comparing whole-chloroplast genome sequences in seed plants. Sci Rep.

[53] Palmer J.D. (1985). Comparative organisation of chloroplast genomes. Annu Rev Genet.

[54] Ku C., Nelson-Sathi S., Roettger M., Garg S., Hazkani-Covo E., Martin William F. (2015). Endosymbiotic gene transfer from prokaryotic pangenomes: Inherited chimerism in eukaryotes. Proc Natl Acad Sci USA.

[55] Stern D.B., Goldschmidt-Clermont M., Hanson M.R. (2010). Chloroplast RNA metabolism. Annu Rev Plant Biol.

[56] Bock R., Bock R. (2007). Structure, function, and inheritance of plastid genomes, in *Cell and Molecular Biology of Plastids*.

[57] Ohyama K. (1996). Chloroplast and mitochondrial genomes from a liverwort, Marchantia polymorpha--gene organization and molecular evolution. Biosci, Biotechnol, Biochem.

[58] del Campo E.M., Sabater B., Martín M. (2000). Transcripts of the ndhH-D operon of barley plastids: possible role of unedited site III in splicing of the ndhA intron. Nucleic Acids Res.

[59] Serrot P., Sabater B., Martín M. (2008). Expression of the ndhCKJ operon of barley and editing at the 13th base of the mRNA of the ndhC gene. Biol Plant.

[60] Neuhaus H.E., Emes M.J. (2000). Nonphotosynthetic metabolism in plants. annu rev plant physio. Plant Mol Biol.

[61] Nixon P.J. (2000). Chlororespiration*.* Philosophical transactions of the Royal Society of London. Series B. Biol Sci.

[62] Casano L.M., Zapata J.M., Martín M., Sabater B. (2000). Chlororespiration and poising of cyclic electron transport. Plastoquinone as electron transporter between thylakoid NADH dehydrogenase and peroxidase. J Biol Chem.

[63] Poetsch A., Rexroth S., Heberle J., Link T.A., Dencher N.A., Seelert H. (2003). Characterisation of subunit III and its oligomer from spinach chloroplast ATP synthase. Biochim Biophys Acta - Biomembr.

[64] Sugimoto K., Okegawa Y., Tohri A., Long T.A., Covert S.F., Hisabori T. (2013). A single amino acid alteration in PGR5 confers resistance to antimycin A in cyclic electron transport around PSI. Plant Cell Physiol.

[65] Zhao J., Yu W., Zhang L., Liu J. (2021). Chlororespiration protects the photosynthetic apparatus against photoinhibition by alleviating inhibition of photodamaged-PSII repair in Haematococcus pluvialis at the green motile stage. Algal Res.

[66] Tan Y., Zhang Q.S., Zhao W., Liu Z., Ma M.Y., Zhong M.Y. (2020). Chlororespiration serves as photoprotection for the photo-inactivated oxygen-evolving complex in zostera marina, a marine angiosperm. Plant Cell Physiol.

[67] Alafari H.A., Abd-Elgawad M.E. (2021). Differential expression gene/protein contribute to heat stress-responsive in Tetraena propinqua in Saudi Arabia. Saudi J Biol Sci.

[68] Li Q., Yao Z.-J., Mi H. (2016). Alleviation of photoinhibition by co-ordination of chlororespiration and cyclic electron flow mediated by NDH under heat stressed condition in tobacco. Front Plant Sci.

[69] Segura M.V., Quiles M.J. (2015). Involvement of chlororespiration in chilling stress in the tropical species Spathiphyllum wallisii. Plant, Cell Environ.

[70] Paredes M., Quiles M.J. (2013). Stimulation of chlororespiration by drought under heat and high illumination in Rosa meillandina. J Plant Physiol.

[71] Rumeau D., Peltier G., Cournac L. (2007). Chlororespiration and cyclic electron flow around PSI during photosynthesis and plant stress response. Plant, Cell Environ.

[72] Cruz J.A., Avenson T.J., Kanazawa A., Takizawa K., Edwards G.E., Kramer D.M. (2005). Plasticity in light reactions of photosynthesis for energy production and photoprotection. J Exp Bot.

[73] Kramer D.M., Avenson T.J., Kanazawa A., Cruz J.A., Ivanov B., Edwards G.E., Papageorgiou G.C., Govindjee (2004). *The Relationship between Photosynthetic Electron Transfer and its Regulation*, in Chlorophyll a Fluorescence: A Signature of Photosynthesis.

[74] Biehler K., Fock H. (1996). Evidence for the Contribution of the Mehler-Peroxidase Reaction in Dissipating Excess Electrons in Drought-Stressed Wheat. Plant Physiol.

[75] Farineau J. (1999). Study of the non-photochemical dark rise in chlorophyll fluorescence in pre-illuminated leaves of various C3 and C4 plants submitted to partial anaerobiosis. Plant Physiol Biochem.

[76] Casano L.M., Martín M., Sabater B. (2001). Hydrogen peroxide mediates the induction of chloroplastic Ndh complex under photooxidative stress in barley1. Plant Physiol.

[77] Martín M., Funk H.T., Serrot P.H., Poltnigg P., Sabater B. (2009). Functional characterization of the thylakoid Ndh complex phosphorylation by site-directed mutations in the ndhF gene. Biochim Biophys Acta - Bioenerg.

[78] Martín M., Casano L.M., Zapata J.M., Guéra A., Del Campo E.M., Schmitz‐Linneweber C. (2004). Role of thylakoid Ndh complex and peroxidase in the protection against photo‐oxidative stress: fluorescence and enzyme activities in wild‐type and ndhF‐deficient tobacco. Physiol Plant.

[79] Lennon A.M., Prommeenate P., Nixon P.J. (2003). Location, expression and orientation of the putative chlororespiratory enzymes, Ndh and IMMUTANS, in higher-plant plastids. Planta.

[80] Baena-González E., Allahverdiyeva Y., Svab Z., Maliga P., Josse E.M., Kuntz M. (2003). Deletion of the tobacco plastid psbA gene triggers an upregulation of the thylakoid-associated NAD(P)H dehydrogenase complex and the plastid terminal oxidase (PTOX). Plant J.

[81] Casano L.M., Martín M., Zapata J.M., Sabater B. (1999). Leaf age- and paraquat concentration-dependent effects on the levels of enzymes protecting against photooxidative stress. Plant Sci.

[82] Guéra A., Sabater B. (2002). Changes in the protein and activity levels of the plastid NADH–plastoquinone–oxidoreductase complex during fruit development. Plant Physiol Biochem.

[83] Martín M., Casano L.M., Sabater B. (1996). Identification of the product of ndhA gene as a thylakoid protein synthesized in response to photooxidative treatment. Plant Cell Physiol.

[84] Lascano H.R., Casano L.M., Martín M., Sabater B. (2003). The activity of the chloroplastic Ndh complex is regulated by phosphorylation of the NDH-F subunit. Plant Physiol.

[85] Sabater B., Martín M. (2013). Hypothesis: increase of the ratio singlet oxygen plus superoxide radical to hydrogen peroxide changes stress defense response to programmed leaf death. Front Plant Sci.

[86] Sabater B., Martín M., Biswal B., Krupinska K., Biswal U.C. (2013). *Chloroplast Control of Leaf Senescence*, in *Plastid Development in Leaves during Growth and Senescence*.

[87] Liu Y., Ren D., Pike S., Pallardy S., Gassmann W., Zhang S. (2007). Chloroplast-generated reactive oxygen species are involved in hypersensitive response-like cell death mediated by a mitogen-activated protein kinase cascade. Plant J.

[88] Heber U., Walker D. (1992). Concerning a dual function of coupled cyclic electron transport in leaves. Plant Physiol.

[89] Haberhausen G., Zetsche K. (1994). Functional loss of all ndh genes in an otherwise relatively unaltered plastid genome of the holoparasitic flowering plant Cuscuta reflexa. Plant Mol Biol.

[90] Luo J., Hou B.-W., Niu Z.-T., Liu W., Xue Q.-Y., Ding X.-Y. (2014). Comparative chloroplast genomes of photosynthetic orchids: insights into evolution of the orchidaceae and development of molecular markers for phylogenetic applications. PLOS ONE.

[91] Lohan A.J., Wolfe K.H. (1998). A subset of conserved tRNA genes in plastid DNA of nongreen plants. Genetics.

[92] Krause K. (2008). From chloroplasts to “cryptic” plastids: evolution of plastid genomes in parasitic plants. Curr Genet.

[93] Bidartondo M.I. (2005). The evolutionary ecology of myco-heterotrophy. N Phytol.

[94] Chang C.C., Lin H.C., Lin I.P., Chow T.Y., Chen H.H., Chen W.H. (2006). The chloroplast genome of Phalaenopsis aphrodite (Orchidaceae): comparative analysis of evolutionary rate with that of grasses and its phylogenetic implications. Mol Biol Evol.

[95] Sanderson M.J., Copetti D., Búrquez A., Bustamante E., Charboneau J.L., Eguiarte L.E. (2015). Exceptional reduction of the plastid genome of saguaro cactus (Carnegiea gigantea): Loss of the ndh gene suite and inverted repeat. Am J Bot.

[96] Lee H., Golicz A.A., Bayer P.E., Jiao Y., Tang H., Paterson A.H. (2016). The genome of a southern hemisphere seagrass species (Zostera muelleri). Plant Physiol.

[97] Williams S.L. (2016).

[98] Olsen J.L., Rouzé P., Verhelst B., Lin Y.-C., Bayer T., Collen J. (2016). The genome of the seagrass Zostera marina reveals angiosperm adaptation to the sea. Nature.

[99] Chen J., Zang Y., Shang S., Liang S., Zhu M., Wang Y. (2021). Comparative chloroplast genomes of zosteraceae species provide adaptive evolution insights into seagrass. Front Plant Sci.

[100] Peltier G., Aro E.M., Shikanai T. (2016). NDH-1 and NDH-2 plastoquinone reductases in oxygenic photosynthesis. Annu Rev Plant Biol.

[101] Scobeyeva V.A., Artyushin I.V., Krinitsina A.A., Nikitin P.A., Antipin M.I., Kuptsov S.V. (2021). Gene loss, pseudogenization in plastomes of genus allium (Amaryllidaceae), and putative selection for adaptation to environmental conditions. Front Genet.

[102] Folk R.A., Sewnath N., Xiang C.-L., Sinn B.T., Guralnick R.P. (2020). Degradation of key photosynthetic genes in the critically endangered semi-aquatic flowering plant Saniculiphyllum guangxiense (Saxifragaceae). BMC Plant Biol.

[103] Ivanova Z., Sablok G., Daskalova E., Zahmanova G., Apostolova E., Yahubyan G. (2017). Chloroplast genome analysis of resurrection tertiary relict haberlea rhodopensis highlights genes important for desiccation stress response. Front Plant Sci.

[104] Zapata J.M., Guéra A., Esteban-Carrasco A., Martín M., Sabater B. (2005). Chloroplasts regulate leaf senescence: delayed senescence in transgenic ndhF-defective tobacco. Cell Death Differ.

[105] Braukmann T.W., Kuzmina M., Stefanović S. (2009). Loss of all plastid ndh genes in Gnetales and conifers: extent and evolutionary significance for the seed plant phylogeny. Curr Genet.

[106] Martín M., Sabater B. (2010). Plastid ndh genes in plant evolution. Plant Physiol Biochem.

[107] Pearson P.N., Palmer M.R. (2000). Atmospheric carbon dioxide concentrations over the past 60 million years. Nature.

[108] Sun Y., Deng T., Zhang A., Moore M.J., Landis J.B., Lin N. (2020). Genome sequencing of the endangered kingdonia uniflora (circaeasteraceae, ranunculales) reveals potential mechanisms of evolutionary specialization. iScience.

[109] Su Y., Huang L., Wang Z., Wang T. (2018). Comparative chloroplast genomics between the invasive weed Mikania micrantha and its indigenous congener Mikania cordata: Structure variation, identification of highly divergent regions, divergence time estimation, and phylogenetic analysis. Mol Phylogen Evol.

[110] Murata N., Takahashi S., Nishiyama Y., Allakhverdiev S.I. (2007). Photoinhibition of photosystem II under environmental stress. Biochim Biophys Acta - Bioenerg.

[111] Niyogi K.K., Truong T.B. (2013). Evolution of flexible non-photochemical quenching mechanisms that regulate light harvesting in oxygenic photosynthesis. Curr Opin Plant Biol.

[112] Li X.P., Björkman O., Shih C., Grossman A.R., Rosenquist M., Jansson S. (2000). A pigment-binding protein essential for regulation of photosynthetic light harvesting. Nature.

[113] Munekage Y., Takeda S., Endo T., Jahns P., Hashimoto T., Shikanai T. (2001). Cytochrome b6f mutation specifically affects thermal dissipation of absorbed light energy in Arabidopsis. Plant J.

[114] McCoy S.R., Kuehl J.V., Boore J.L., Raubeson L.A. (2008). The complete plastid genome sequence of Welwitschia mirabilis: an unusually compact plastome with accelerated divergence rates. BMC Evol Biol.

[115] James D.A. (1983).

[116] Bassam N.E. (2010). Development and Applications.

[117] Matthews R.F., Fire Effects Information System. 1994, U.S. Department of Agriculture, Forest Service, Rocky Mountain Research Station, Fire Sciences Laboratory (Producer). p. https://www.fs.fed.us/database/feis/plants/shrub/simchi/all.html.

