# Supplementary material for: Loss of plastid ndh genes in an autotrophic desert plant
Source: Comput Struct Biotechnol J. 2023 Oct 14;21:5016–27. doi: 10.1016/j.csbj.2023.10.023 (PMC10589726; doi:10.1016/j.csbj.2023.10.023)
Supplement: Supplementary file 1 — Supplementary material. [file mmc1.docx]

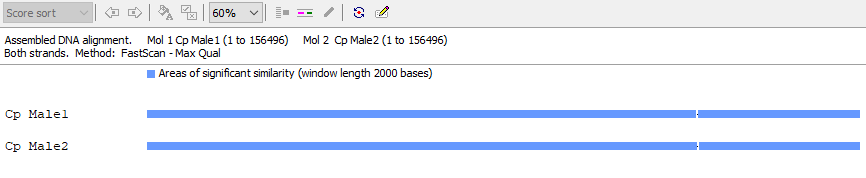


**JmaleCp1**

**JmaleCp2**


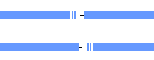


**Supplementary Figure 1:** **Alignment of two GetOrganelle-derived plastome (JmaleCp1 and JmaleCp2) sequences from a male Jojoba plant**

The two plastome sequences were the same size (156,496 bp) with complete match except for one region covering 1,261 bp and shown as a bold red line. The mismatch region is caused because the 1,261 bp sequence in JmaleCp1 is inverted in JmaleCp2.


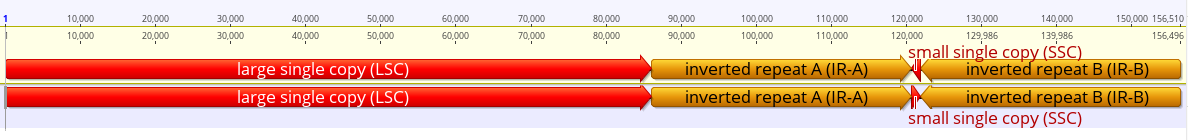


**Jojoba Male Cp2 (JmaleCp2)**

**Jojoba Male Cp1 (JmaleCp1)**

**Supplementary Figure 2:** **GeSeq derived structural annotation of the two jojoba male plastome sequences.**

The two jojoba plastome (Cp) sequences (156,496 bp), jojoba Male Cp1 (JmaleCp1) and jojoba Male Cp2 (JmaleCp2), have the same length and structural annotation comprising of LSC (85,995 bp), IR-A and IR-B (each at 34,620 bp), except for the mismatch region of 1,261 bp annotated to be the Small Single Copy (SSC). The orientation of the SSC in JmaleCp1 and JmaleCp2 are in opposite direction. In JmaleCp1, the orientation of the LSC and IR-A regions is opposite to that of the SSC and IR-B regions. In JmaleCp2, the orientation of the LSC, IR-A and SSC regions is opposite to that of the IR-B region. in In JmaleCp-2, the orientation of LSC, IR-A, SSC and IR-B (>LSC>IR-A>SCSC<IR-B) matches the usually adopted orientation when depicting a linear plastome sequence.


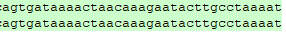


**112,608 bp**

**129,884 bp**


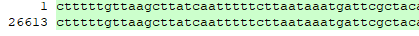


**85,996 bp**

**156,496 bp**

**B**


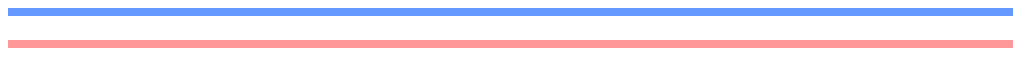


**Jojoba part IR-A**

**Jojoba part IR-B**


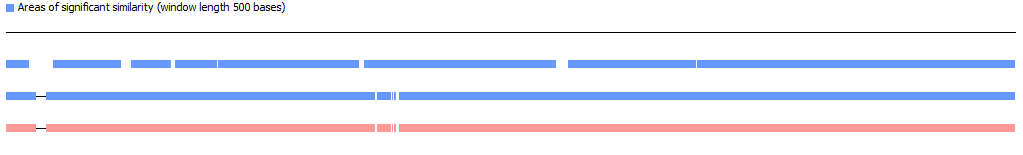


**26,613 bp**


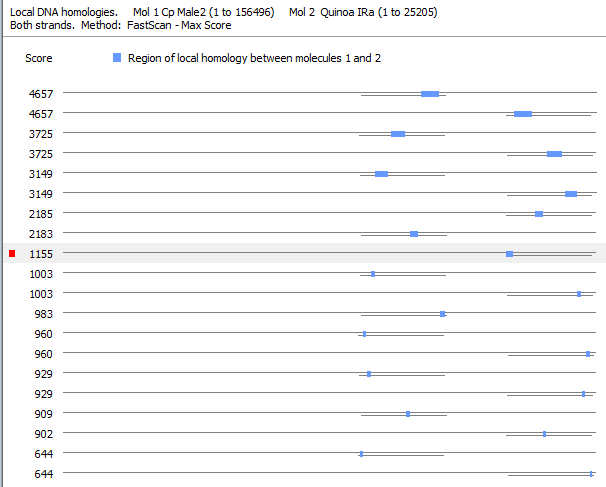

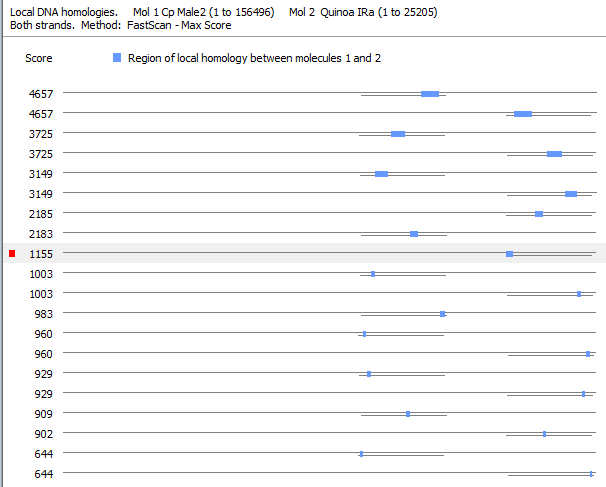


**A**

**Alignment 1**

**Alignment 2**

**Jojoba IR-A 34,620 bp**

**Quinoa IR**

**Jojoba IR-B 34,620 bp**

**Quinoa IR**

Local DNA homologies:

Molecule 1: JojobaCp, Molecule 2: QuinoaCp IRA (1 to 25,205 Both Strands)

**8,007 bp**

**8,007 bp**

**C**


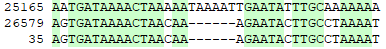


**108,787 bp**

**112,608 bp**

**129,884 bp**


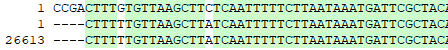


**83,583 bp**

**85,996 bp**

**156,496 bp**

**Quinoa IR**

**Jojoba part IR-A**

**Jojoba part IR-B**


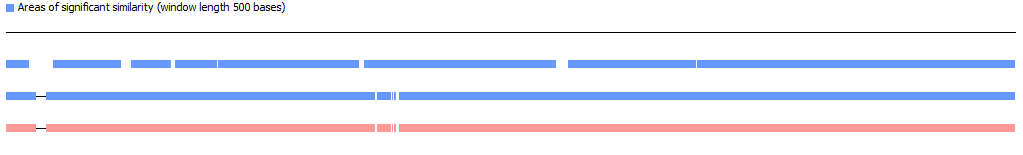

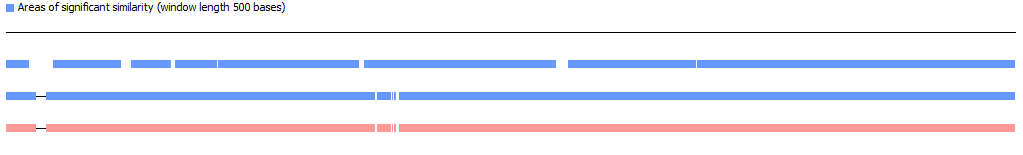


**26,613 bp**

**25,205 bp**

**26,613 bp**

**26,613 bp**

**Supplementary Figure 3: Similarity between the IR regions of the jojoba and quinoa plastome sequences (JojobaCp and QuinoaCp).**

IR-A and IR-B, Inverted repeat A and B respectively; QuinoaCp, Chenopodium quinoa (KY419706); blue/red horizontal lines with arrow ends or with filled circle end, GeSeq-annotated IR region of the JojobaCp or region of the Jojoba IR region matching Quinoa IR region, respectively; black line square boxes, additional region (8,007bp) of the JojobaCp IR region not matching the Quinoa IR; part IR-A and part IR-B, Jojoba IR region matching the Quinoa IR region; A, Sequence alignment between the IR sequence of the JojobaCp (longer sequence) and the QuinoaCp (shorter sequence); B, Sequence alignment of the Jojoba part IR-A and part IR-B; C, Sequence alignment between the Quinoa IR region and the Jojoba part IR-A and part IR-B. The Ge-Seq annotated JojobaCp sequence indicates an expanded IR region of 34,620 and consists of a region of 26,613 bp similar to the QuinoaCp IR region (25,205 bp) and an additional region of 8,007 bp as identified by black line boxes.


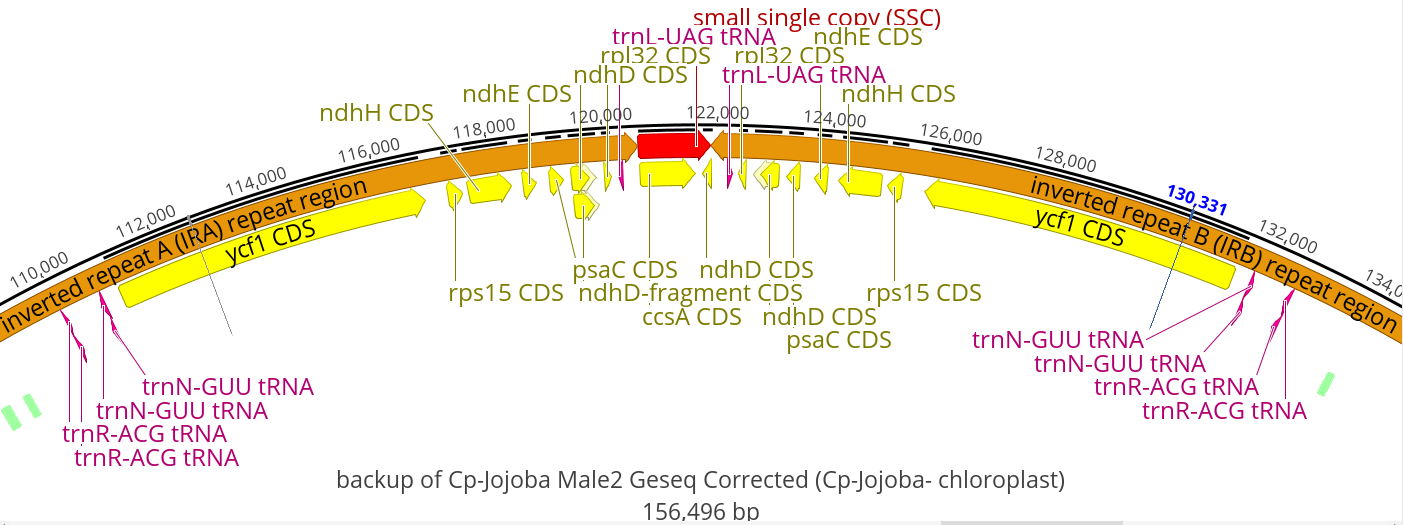


***Simmondsia chinensis* Plastome**

**(JojobaCp, 156,496 bp)**

**IR-A region (8,007 bp) with Part SSC genes**

**IR-**B **region (8,007 bp) with Part SSC genes**

**SSC**

**(1,261 bp)**

**B**


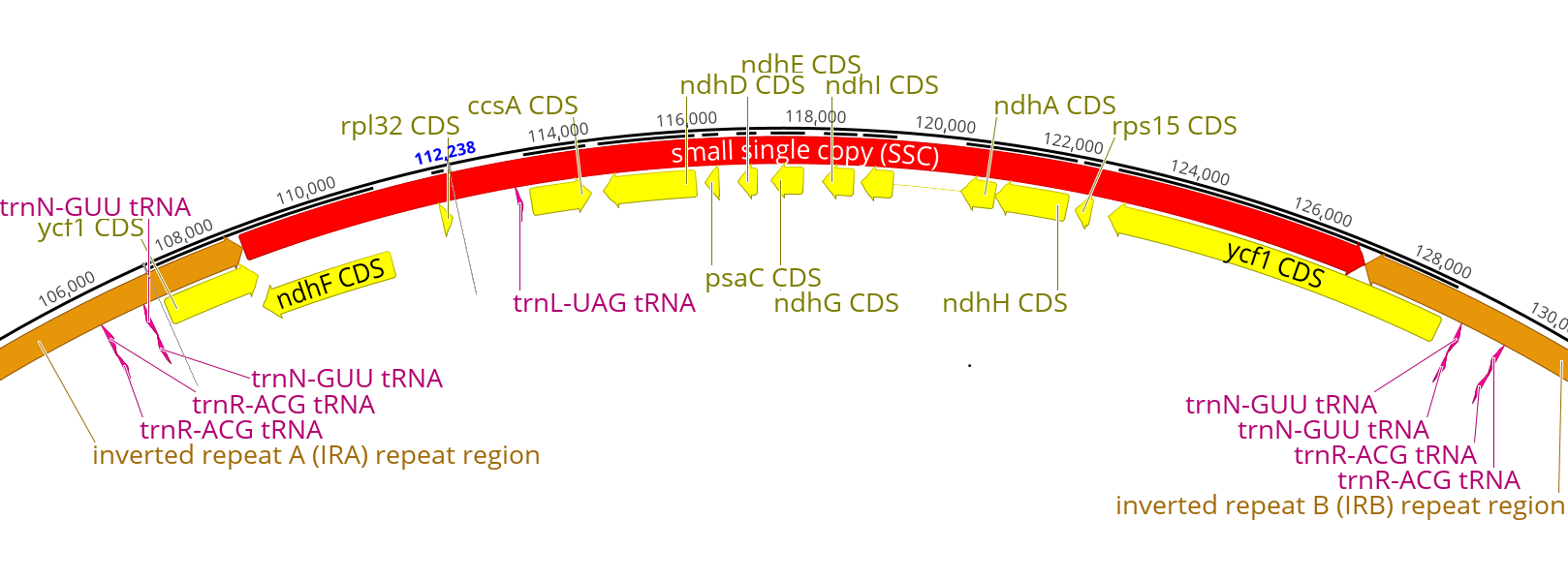


**A**

***Chenopodium quinoa* Plastome**

**(QuinoaCp, 152,099 bp)**

**Supplementary Figure 4:** **Comparison of Small single copy (SSC) and Inverted Repeat regions of the jojoba plastome and the quinoa plastome (KY419706)**

**A**, **B**, GeSeq annotated regions of the plastome sequences of *Chenopodium quinoa* (QuinoaCp) and *Simmondsia chinensis* (JojobaCp). The 1,261 bp SSC region of the JojobaCp has only the *ccsA* CDS. Genes on the IR-A and IR-B regions on the JojobaCp identified by blue-bordered boxes and contiguous to the left and right respectively to the jojoba SSC region, are also present on the SSC region on the QuinoaCp sequence. Sequences within the blue-bordered boxes are identical (8,007 bp) and inverted repeats of each other and labelled as “Part- SSC” (P-SSC) and in between these lies the 1,261 bp SSC region. The *ccsA* CDS gene in the SSC region and the genes within the P-SSC on IR-B are in the same orientation as found in *C. quinoa* as indicated in bold blue arrow.

**QuinoaCp SSC**

**JojobaCp SSC**

**JojobaCp P-SSC**


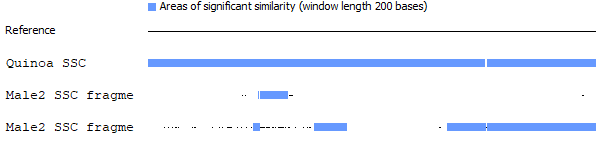


po

**Supplementary Figure 5:** **Alignment showing regions of the QuinoaCp SSC region missing in the JojobaCp SSC or the P-SSC regions.**

Quinoa (*Chenopodium quinoa*); Jojoba (*Simmondsia chinensis);* Cp, plastome; SSC, small single copy; P-SSC, Part of the SSC now part of the inverted Repeat region. Regions of the QuinoaCp SSC region missing in the in the JojobaCp SSC and the JojobaCp P-SSC regions are indicated as black bordered boxes.


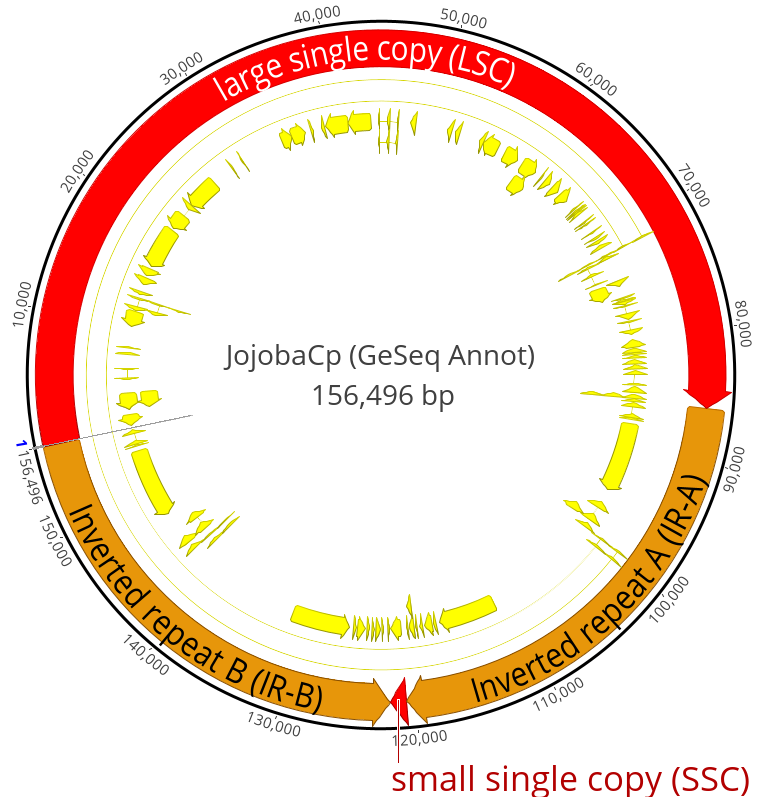


**A**


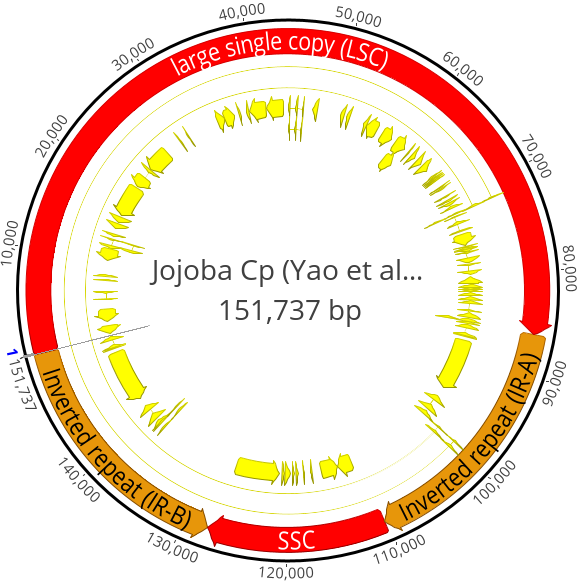


**B**

**Supplementary Figure 6: Graphical representation of the jojoba plastome sequence and that reported by Yao et al 2019.**

**A** , JojobaCp; **B**, JojobaCp (reported by Yao et al., 2019, NC 040935); **A and B**, GeSeq annotation and Geneious-generated map; SSC, small single copy; Cp, plastome. Differences between the JojobaCp sequence and that reported by Yao et al. includes a larger JojobaCp sequence (156, 496 bp vs 151,737 bp) with an expanded IR sequences (34,620 bp vs 24,082 bp) and a smaller SSC sequence (1,261 bp vs 17,367 bp). Annotations of both plastomes were undertaken by GeSeq.


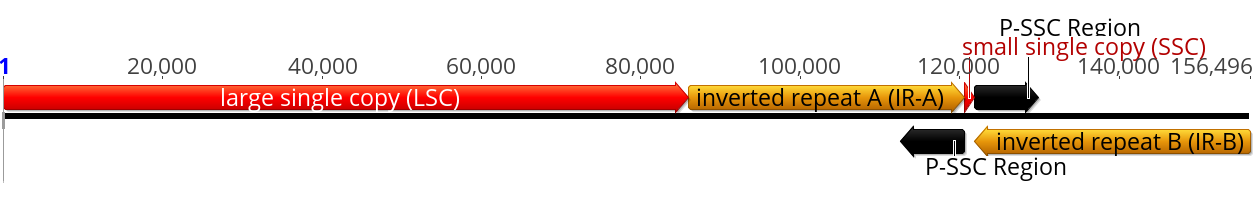

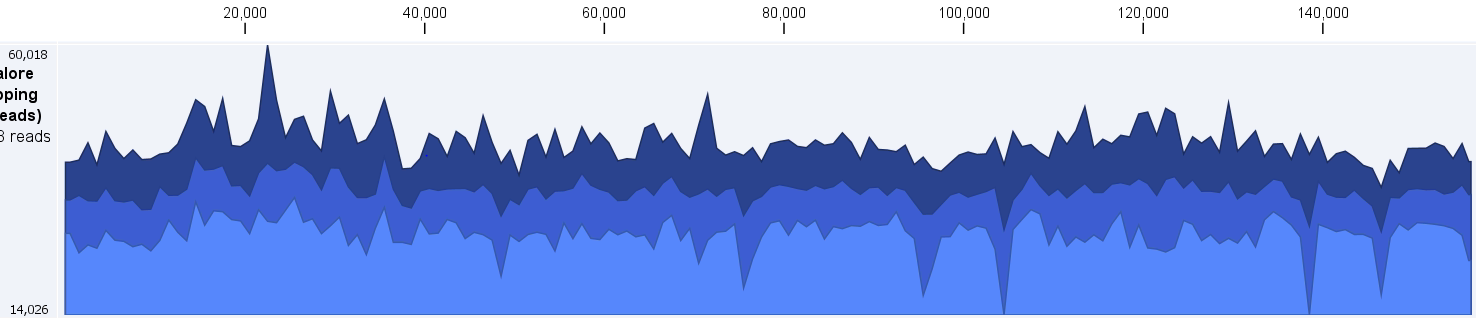


**A**

**JojobaCp 156,496 bp**


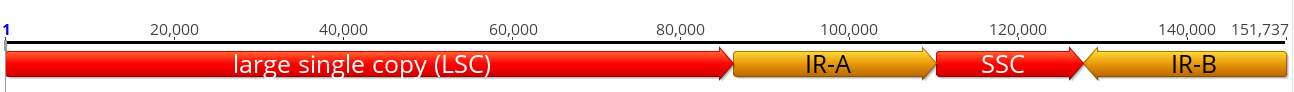

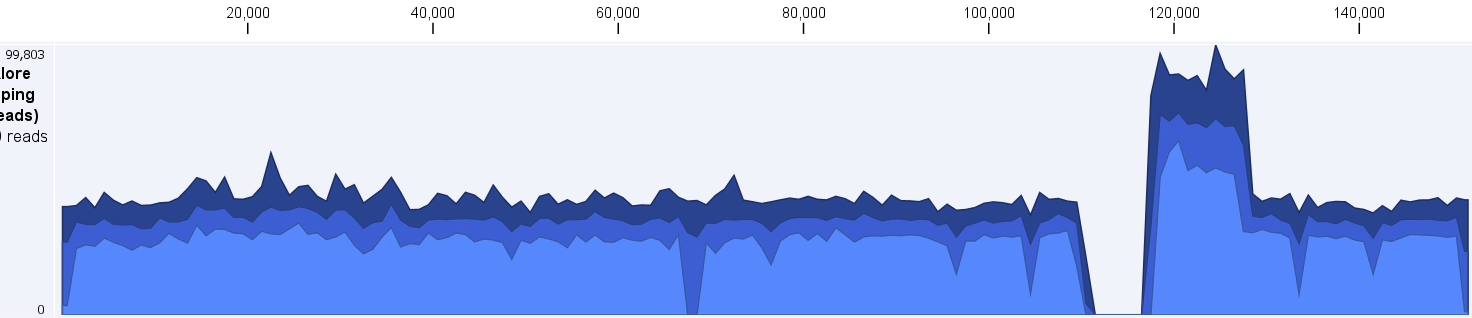


**B**

**Yao JojobaCp (Yao et al. 2019) 151,737 bp**

**Supplementary Figure 7: Mapping coverage of illumina reads to two jojoba plastome sequences.**

**A**, mapping coverage to the JojobaCp as reference; **B**, mapping coverage to the Yao JojobaCp NC_040935 (Yao et al 2019 DOI: 10.1016/j.ympev.2018.12.023) as reference. Whole genome illumina reads (150bp aired end reads) from the Jojoba Male genotype were mapped at high coverage throughout the JojobaCp sequences and at high coverage throughout the Yao JojobaCp (Yao et al. 2019) except part of the SSC region. Graph tracks are composed of three curves showing the maximum, mean, and minimum coverage value observed in a given region.


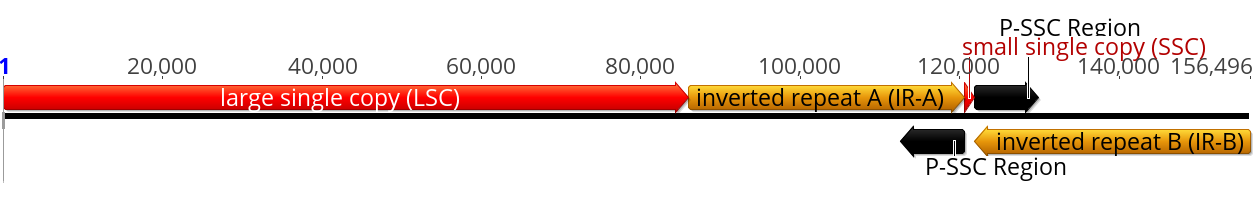


**JojobaCp 156,496 bp**


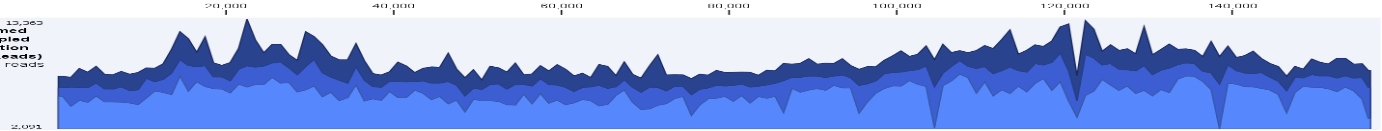


**A**


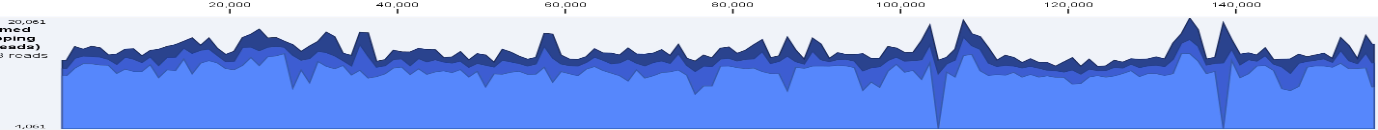


**E**


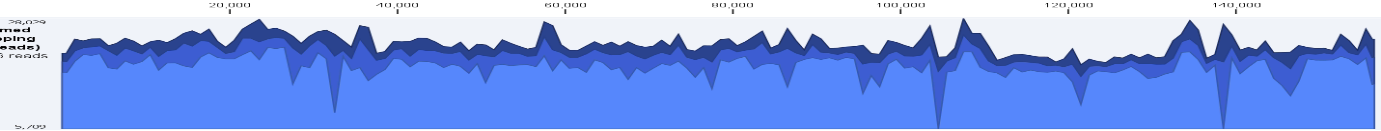


**D**


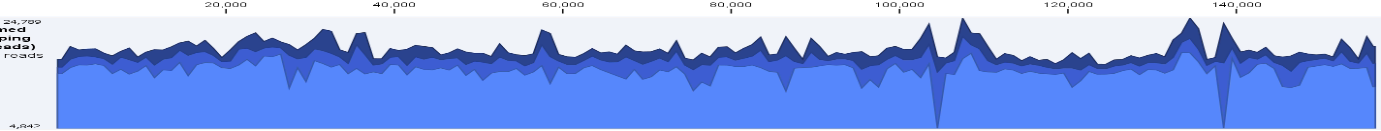


**C**


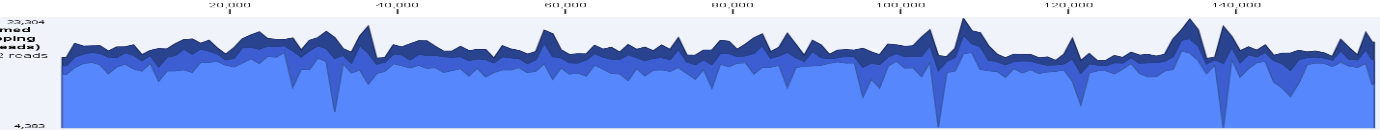


**B**

**Yao JojobaCp 151,737 bp**


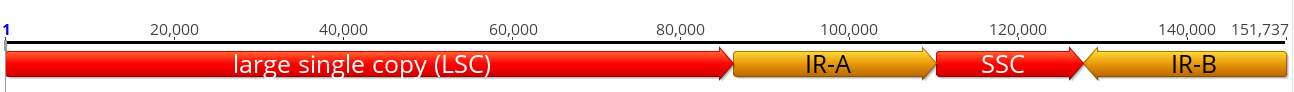

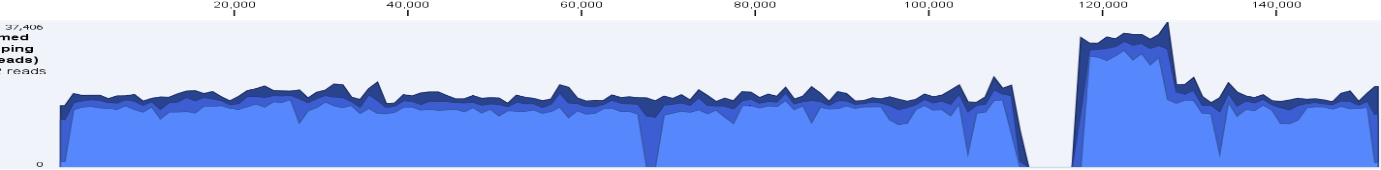


**G**


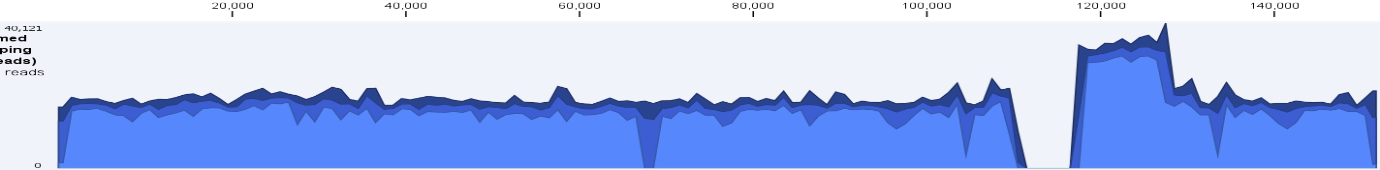


**H**


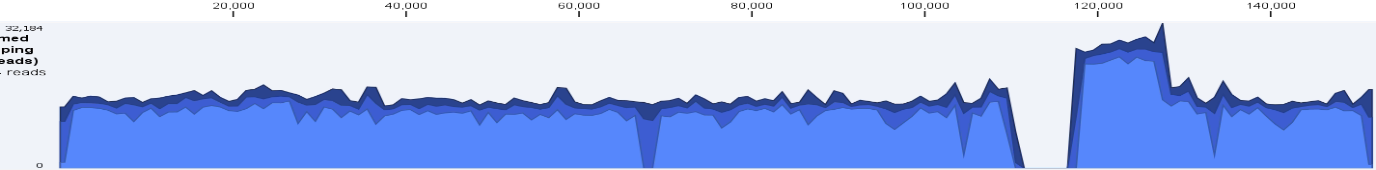


**I**


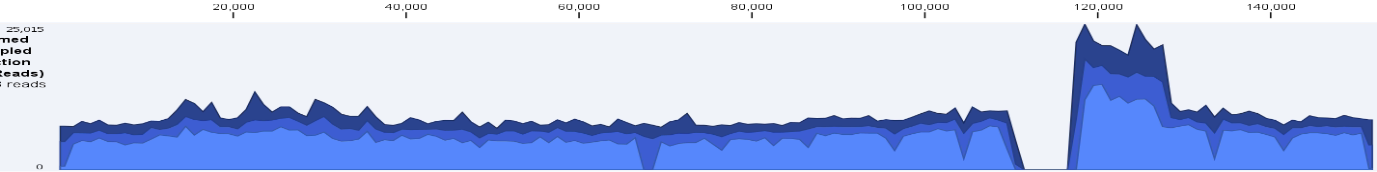


**F**


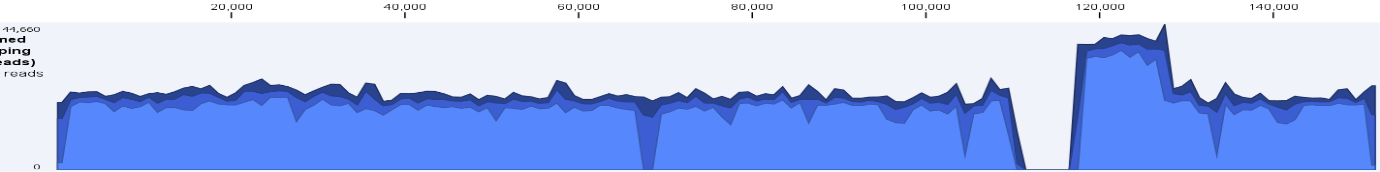


**J**

**Supplementary Figure 8: Mapping coverage against two jojoba plastome sequences using reads from several male and female jojoba accessions.**

**A,F**, Jojoba female; **B,G**, Jojoba Male Dadi-dadi; **C,H,** Jojoba Male T100; **D,I,** Jojoba Female Wadi-wadi; **E,J,** Jojoba Female Q103; **A,B,C,D,E**, Mapping of reads against the JojobaCp sequence; **F,G,H,I,J,** YaoJojobaCp. Whole genome illumina reads mapped at high coverage throughout the jojoba plastome sequences (**A to E**) and also at high coverage throughout the YaoJojobaCp except part of the SSC region (**F to J**). Graph tracks are composed of three curves showing the maximum, mean, and minimum coverage value observed in a given region.


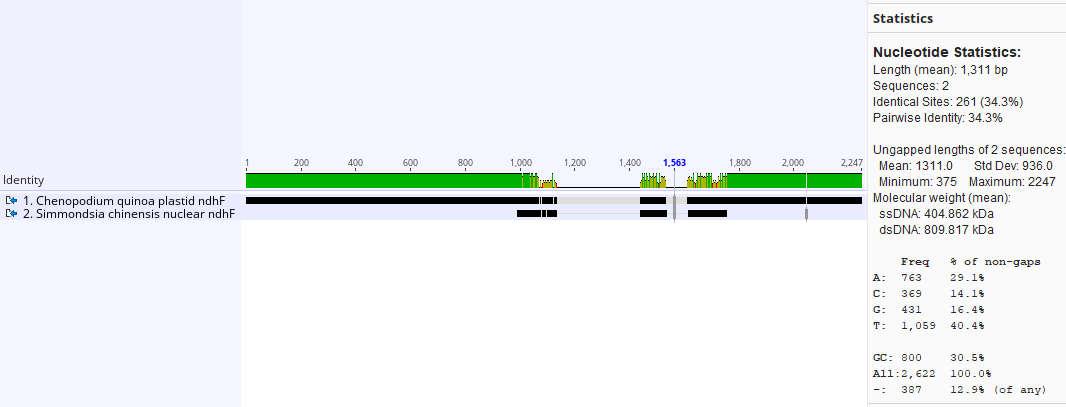


**Supplementary Figure 9: Alignment of the *ndhF* gene sequence sequences from quinoa plastome and the jojoba nuclear genome**

Alignment of the nuclear *Simmondsia chinensis* (Jojoba) nuclear *ndhF* gene (375 bp) and the *Chenopodium quinoa* plastid *ndh*F gene (2,247 bp), shows 34.3% identity over 261bp. Pairwise alignment was undertaken using Clustal Omega (version 1.2.2) available in Geneious software.
